# Pharmacogenomics as a Tool to Limit Acute and Long-Term Adverse Effects of Chemotherapeutics: An Update in Pediatric Oncology

**DOI:** 10.3389/fphar.2020.01184

**Published:** 2020-08-05

**Authors:** Emma C. Bernsen, Melanie M. Hagleitner, Theodorus W. Kouwenberg, Lidwien M. Hanff

**Affiliations:** ^1^ Pharmacy, Princess Máxima Centre for Pediatric Oncology, Utrecht, Netherlands; ^2^ Department of Pediatric Hemato-oncology, Princess Máxima Centre for Pediatric Oncology, Utrecht, Netherlands

**Keywords:** pediatric oncology, chemotherapeutic agents, drug toxicity, adverse effects, pharmacogenomics

## Abstract

In the past decades, new cancer treatments have been introduced in pediatric oncology leading to improvement in clinical outcomes and survival rates. However, due to inter-individual differences, some children experience severe chemotherapy-induced toxicities or a poor clinical outcome. An explanation for the diversity in response to chemotherapy is genetic variation, leading to differences in expression and activity of metabolizing and transport enzymes as well as drug targets. Pharmacogenetic testing has emerged as a promising tool to predict and limit acute and long-term adverse effects in patients. However, in pediatric oncology, limited number of patients and a considerable diversity in study results complicate the interpretation of test results and its clinical relevance. With this review, we provide an overview of new developments over the past four years regarding relevant polymorphisms related to toxicity in pediatric oncology. The following chemotherapeutics and associated toxicities are discussed: alkylating agents, anthracyclines, asparaginase, methotrexate, platinum compounds, steroids, thiopurines, topoisomerase inhibitors, and vinca alkaloids. Our review identifies several questions regarding the role of genetic variants in chemotherapy-induced toxicities. Ambiguities in the literature stem from small population sizes, differences in (statistical) interpretation and variations in sequencing technologies as well as different clinical outcome definitions. Standardization of clinical outcome data and toxicity definitions within electronic health records combined with the increased availability of genomic sequence techniques in clinical practice will help to validate these models in upcoming years.

## Introduction

Over the past decades, the 5‐year survival rate for childhood cancer improved from 58% for children diagnosed during 1975 to 1977 to 83% for those diagnosed during 2005 to 2015 (O’Leary et al., 2008; Pui et al., 2011; Siegel et al., 2019). This improvement is mainly driven by risk stratification and intensification of cytotoxic chemotherapy. As survival is increasing, the focus has shifted to decreasing serious toxicities of chemotherapy without losing anti-tumor effectiveness of multimodality treatment. Inter-individual differences in drug response have been implicated as an important consequence of chemotherapy-induced toxicities (Roden et al., 2019). As a result, genetic predisposition has been proposed as an explanation for individual variation in chemotherapeutic response and toxicity.

Pharmacogenomics includes all studies assessing genetic variations in patients influencing pharmacokinetics (drug absorption, metabolism, excretion, cellular transport) and pharmacodynamics. Pharmacogenetic testing has emerged as a promising tool to predict and limit acute and long term adverse effects in individual patients, and is widely investigated in pharmacogenomics (PGx) and genome-wide association studies (GWAS) (Relling et al., 2020). At present, the Clinical Pharmacogenetics Implementation Consortium (CPIC) has provided guidelines for the implementation of pharmacogenetics in practice, which led to 23 clinical guidelines, comprising 19 genes and 46 drugs (Haidar et al., 2019; Relling et al., 2020). In the Netherlands, The Dutch Pharmacogenetics Working Group (DPWG) has reviewed 97 gene-drug interactions, leading to multiple recommendations for clinical practice (KNMP apothekersorganisatie; van der Wouden et al., 2019). However, these guidelines are predominantly based on research in adults and exclude some pediatric cancer treatment drugs (e.g. asparaginase).

The rarity of childhood cancer and inherently small patient populations combined with diversity in outcome measurements led to uncertainties regarding the clinical relevance of genetic testing in pediatric oncology as well as difficulties with the interpretation of test results (Relling and Klein, 2011). Given that high survival rate in pediatric cancer is dependent on intensive chemotherapy, clinicians are hesitant to preemptively reduce dosages due to genetic variants, as retaining the beneficial outcome is of utmost importance in pediatric cancer.

The availability and affordability of genomic technologies have improved greatly in the past years, resulting in many studies, albeit of varying quality. In this literature review we provide an overview of developments and new insights over the last years regarding relevant genes and polymorphisms as well as their role in acute and long-term adverse effects of drugs in pediatric oncology. The review is limited to pediatric age group (0–18 years). To address the recent developments, we reviewed recent publications (from 2016 onward) using terms related to pediatric oncology, pharmacogenomics and pharmacogenetics, and toxicities of (pediatric) cancer drugs. While a wide variety of supportive care drugs (e.g. anti-infective drugs, analgesics drugs, and antiemetic drugs) and immunotherapy are also used in pediatric oncology, we focus exclusively on the association between genetic variants and chemotherapy-induced toxicities.

## Role of Pharmacogenetic Variations in Chemotherapeutic Related Toxicities

The following chemotherapeutics and their toxicities have been selected based upon extensive use in pediatric oncology: alkylating agents, anthracyclines, asparaginase, methotrexate, platinum compounds, steroids, thiopurines, topo-isomerase inhibitors, and vinca alkaloids. For each chemotherapeutic agent, the relationship between various genetic variations and chemotherapy-induced toxicity are discussed. An overview of recent studies included in this review can be found in **Supplementary Table 1**.

### Alkylating Agents

Alkylating agents are widely used antitumor prodrugs deriving their cytotoxic effect from adding an alkyl group to the guanine base of the DNA molecule. This alkylation results in abnormal nucleotide sequences, miscoding of messenger RNA, blockade of DNA replication and breakage of DNA strands and eventually tumor cell death. Most common alkylating agents in pediatric oncology are nitrogen mustards like cyclophosphamide and ifosfamide, alkyl sulfonates like busulfan and triazenes such as dacarbazine and temozolomide. Alkylating agents show a wide range of toxicity including myelosuppression, kidney and gastrointestinal toxicities. Due to limited data, only cyclophosphamide, ifosfamide, and busulfan are updated in this review. In addition to toxicity studies, we also included studies analyzing the association between pharmacokinetics of alkylating agents and genetic variants, while limited studies were available which directly investigated the association between genetic variants and alkylating-induced toxicities.

#### Cyclophosphamide

##### Metabolism and Transport

Activation of cyclophosphamide is catalysed by the hepatic cytochrome P450 (CYP) isozymes *CYP2B6, CYP2C19*, and *CYP3A4*. The overall metabolism of cyclophosphamide is complex, with numerous enzymes involved which vary in expression and activity.

##### Genetic Variances and Toxicity

In the past, *CYP2B6* and *CYP2C19* have shown to influence cyclophosphamide pharmacokinetics in adult patients (Helsby et al., 2010). Recently, the influence of *CYP2B6* on cyclophosphamide clearance was confirmed in the pediatric population of 49 B-cell Non Hodgkin Lymphoma (NHL) patients. Patients carrying CYP2B6*6 had significant lower cyclophosphamide clearance (Veal et al., 2016). This is in line with previous research showing a decreased function of CYP2B6*6 (Lang et al., 2001; Hesse et al., 2004; Zukunft et al., 2005; CYP2B6 P, 2020).

#### Ifosfamide

##### Metabolism and Transport

Ifosfamide requires activation by *CYP3A4* and *CYP2B6* to active metabolites. Variation in the renal expression of *CYP2B6* leads to higher rates of ifosfamide metabolite chloroacetaldehyde (CAA), which is nephrotoxic. Increasing evidence suggests that CAA is also involved in ifosfamide-induced encephalopathy.

##### Genetic Variances and Toxicity

Very limited data is available regarding the influence of genetic variants on toxicity of ifosfamide. CYP2B6*6 carriers have been linked with ifosfamide-induced encephalopathy in a report of three pediatric cases (Duflot et al., 2018). Earlier, this genotype has been linked with lower catalytic activity and protein expression in the liver, higher concentrations of ifosfamide and higher rates of CAA associated toxicity (Wang and Tompkins, 2008). This could be a mechanism for ifosfamide-induced encephalopathy, though more extensive studies are needed to confirm this assumption.

In conclusion, prospective studies are needed to further elucidate the role of CYP2B6 polymorphism in the metabolism and toxicity of cyclophosphamide and ifosfamide.

#### Busulfan

##### Metabolism and Transport

Busulfan, widely used in conditioning regimens before hematopoietic stem cell transplantation, has a narrow therapeutic window and demonstrates wide interpatient variability in pharmacokinetics. High drug exposure is associated with increased risk of toxicities, such as veno-occlusive disease, while low drug exposure is associated with treatment failure. Busulfan is metabolized in the liver by glutathione S-transferase isoenzymes (*GSTs*). *GSTA1* is the predominant GST isoenzyme in the metabolism of busulfan. *GSTM1* and *GSTT1* are involved to a lesser extent.

##### Genetic Variances and Toxicity

In the past, several studies in adult and pediatric patients showed a higher busulfan clearance in patients with GSTA1*A/*A genotype (with consequent lower AUC), while patients with GST*B/*B genotype had lower clearance (with consequent higher AUC) (Myers et al., 2017). While this association has been found, it is noteworthy that not all studies found clinical correlations. Recently, one study has successfully incorporated *GSTA1* genotype into a pharmacokinetic model for busulfan in a group of 112 pediatric patients. In this study, GSTA1*A2 or *A3 homozygote or heterozygote carriers showed a 7% higher clearance. Also, clearance of patients carrying GSTA1*B1b*B1b was 12% lower. Based doses in this study resulted in a better achievement of AUC targets (see **Supplemental Material** of Nava et al. for gene expression information) (Nava et al., 2018). However, another recent study showed no significant association with *GST* polymorphisms and busulfan pharmacokinetics (Nishikawa et al., 2019). These contradictory data may be attributed due to small study cohorts and variation in study design. Further basic research and clinical investigative efforts are required to fully understand the key factors determining busulfan PGx characteristics (Myers et al., 2017).

### Anthracyclines

Anthracyclines are widely used in many pediatric cancers, including leukemia, lymphomas, and solid tumors. Anthracyclines agents are doxorubicin, daunorubicin, idarubicin, epirubicin, and mitoxantrone. While their mechanism of action is not fully known, it is believed anthracyclines interfere with DNA metabolism (including inhibition of topoisomerase II) and damage DNA through reactive oxygen species (ROS) (McGowan et al., 2017; Anthracyclines and related substances, 2020). Notorious for their severe cardiotoxicity, anthracycline cumulative doses are monitored closely during treatment.

#### Anthracycline-Induced Cardiotoxicity

**Figure 1:**
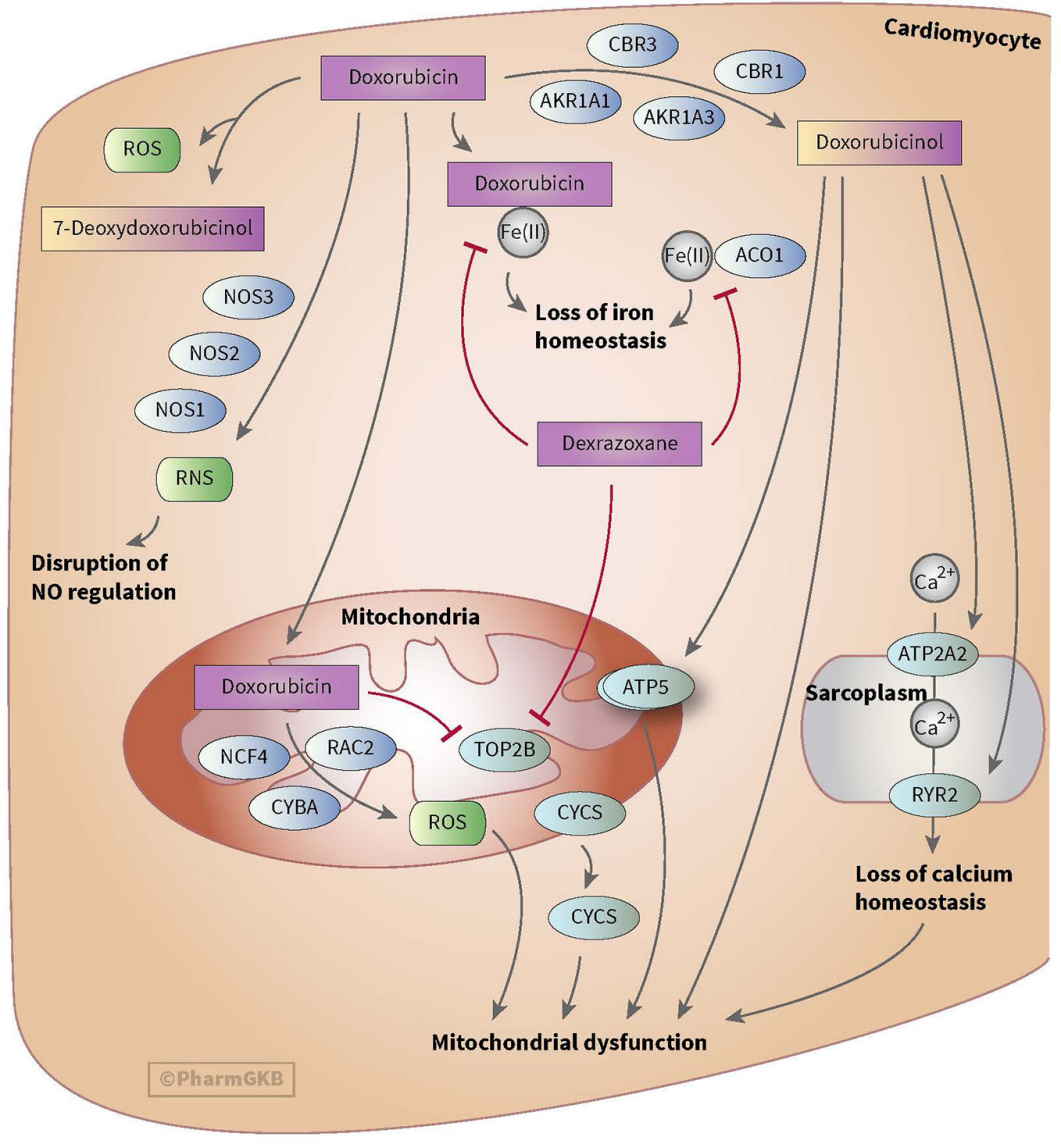
Figure used with permission of PharmGKB (Thorn et al., 2011; Doxorubicin Pathway (Cardiomyocyte Cell) P, 2020). This is an example of genes involved in doxorubicin-induced cardiotoxicity. The presented genes (e.g. CBR, NOS, and AKR) are involved in the metabolizing and transport pathways of anthracyclines. The formation of ROS during the metabolism of anthracyclines is thought to play an important role in anthracycline-induced cardiotoxicity.

**Figure 2:**
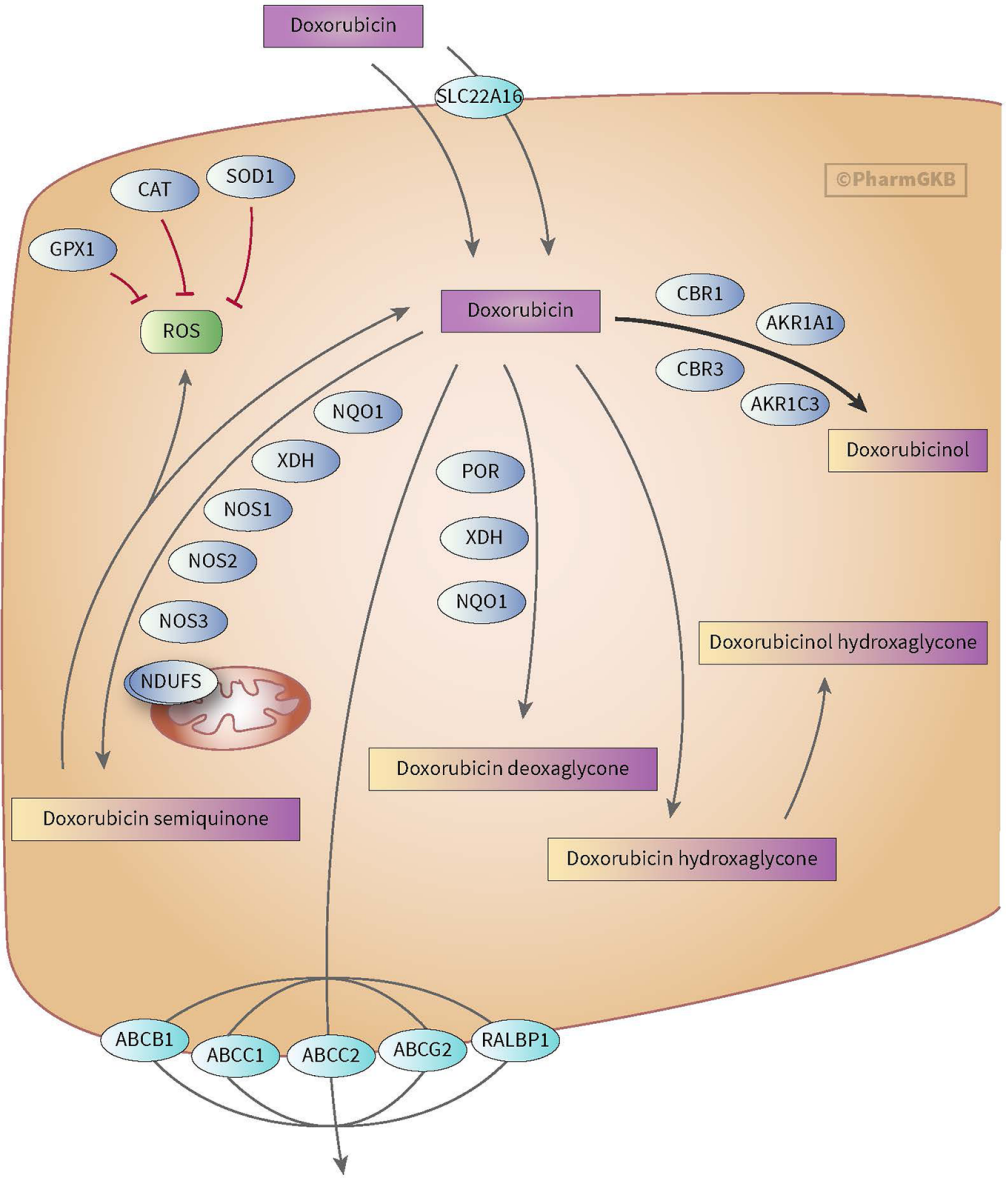
Figure used with permission of PharmGKB (Thorn et al., 2011; Doxorubicin Pathway, 2020). This is an example of genes involved in doxorubicin pharmacokinetic pathway. These include metabolizing genes such as CBR, AKR, and NOS as well as SLC and ABC transporters.

Anthracycline-induced cardiotoxicity can be acute and reversible (within the first weeks of treatment) or develops one or more year(s) after treatment discontinuation and causes chronic cardiotoxicity. There are various theories with regard to the development of anthracycline-induced cardiotoxicity. One theory discusses the formation of ROS and topoisomerase II alterations which causes damage to cardiomyocytes and mitochondria in cells. ROS is mainly formed during anthracyclines metabolism. Also, risk factors such as sex, age, comorbidities, and cumulative dose of anthracyclines (>350 mg/m^2^) play a relevant role in anthracycline-induced cardiotoxicity (see for more in-depth information on cardiotoxicity Bansal et al.’s review) (Bansal et al., 2017). However, ROS formation, topoisomerase II alterations and the mentioned risk factors do not fully explain the inter-individuals differences in the severity of cardiotoxicity among children (Huang et al., 2017). This led to the assumption that genes involved in metabolism and transport of anthracyclines as well as genes associated with the prevention of ROS and genes involved in iron homeostasis could also influence anthracycline-induced cardiotoxicity (see **Figures 1** and **2**) (Thorn et al., 2011; Aminkeng et al., 2016; Doxorubicin Pathway, 2020; Doxorubicin Pathway (Cardiomyocyte Cell) P, 2020).

#### Metabolism and Transport

Anthracyclines are metabolized through three pathways: hydroxylation, semiquinone formation, and/or deoxyaglycone formation. Hydroxylation is mediated by NADPH-dependent carbonyl (*CBR*) and aldo-keto (*AKR*) reductases. Enzymes known for catalyzing semiquinone formation are CYP reductase (*CYPR*), NADH dehydrogenase (*NDUFS*), nitric oxide synthase (*NOS*), and xanthine oxidase. These conversions also increase the formation of reactive oxygen species (ROS). The final metabolizing step is deoxyaglycone formation. This is catalyzed by NADPH quinone oxidoreductases (*NQO1*), *CPR*, and xanthine dehydrogenase (*XDH*) (see **Figures 1** and **2**) (Thorn et al., 2011; Edwardson et al., 2015; Doxorubicin Pathway, 2020; Doxorubicin Pathway (Cardiomyocyte Cell) P, 2020).

While the transport of anthracyclines is not fully known, acknowledged genes involved in transport of anthracyclines are ATP-binding cassette (*ABC*) *ABCB1, ABCC1, ABCC2, ABCG2*, and solute carrier (SLC) *SLC22A16* (see **Figures 1** and **2**).

#### Genetic Variants in Metabolizing and Transport Enzymes

Past studies showed a strong association between genetic variations *SLC28A3* (rs7853758 and rs885004) and detoxifying uridine diphosphate glucuronosyl transferase (UGT) UGT1A6*4 (rs17863783) and anthracycline-induced cardiotoxicity (Visscher et al., 2013; Aminkeng et al., 2016). Also genetic variations in *CBR3* (Blanco et al., 2012; Visscher et al., 2012), *ABCC1* (rs3743527, rs246221) (Blanco et al., 2008), *SLC28A3* (rs885004 and rs4877847), *SLC22A17* (rs4982753, rs4149178) (Visscher et al., 2015), and sulfotransferase (*SULT) SULT2B1* (rs12882406 and rs12896494) (Visscher et al., 2015) have been associated with cardiotoxicity during anthracycline treatment (Conyers et al., 2018). In recent years, three studies have focused on *ABCB1, ABCC1, ABCC2, ABCC5, ABCG2, SLC28A3*, and *CYP3A5* (Krajinovic et al., 2016; Huang et al., 2017; Ruiz-Pinto et al., 2017).

A small Chinese study including 36 ALL patients showed no association between potential metabolizing *CYP3A* polymorphisms (CYP3A5*1‑*1, CYP3A5*1‑*3, CYP3A5*3‑*3) and daunorubicin-induced cardiotoxicity (Huang et al., 2017). A study by Ruiz-Pinto et al. (2017) performed a GWAS with 93 pediatric cancer patients who had used doxorubicin, daunorubicin or epirubicin in the past. In this study, no association was found with transporter *ABCB1* and *SLC28A3* polymorphisms and chronic anthracycline-induced cardiotoxicity. A retrospective cohort study by Krajinovic et al. (Krajinovic et al., 2016) included 251 cALL patients and 44 cALL patients (validity set). Multiple polymorphisms in transporter genes *ABCC1, ABCC2, ABCC5, ABCB1*, and *ABCG2* were investigated for associations with doxorubicin-induced cardiotoxicity. The *ABCC5* (rs7627754) was found to be significant associated with a lower ejection fraction (EF) and shortening fraction (SF) 3 years after diagnosis, suggesting a possible higher risk for cardiotoxicity with patients carrying this polymorphism.

#### Genetic Variants Involved in ROS Prevention

Genetic variants involved in ROS prevention have received much attention in past studies (Aminkeng et al., 2016; Bansal et al., 2017; Chang and Wang, 2018). These include genetic variations in NADPH oxidase, Ras-related C3 botulinum toxin substrate 2 (*RAC2)* (Aminkeng et al., 2015), neutrophil cytosolic factor 4 (*NCF4)* (Visscher et al., 2012), Cytochrome B-245 Alpha Chain (*CYBA)* (Visscher et al., 2012; Windsor et al., 2012; Armenian et al., 2013) and catalase *(CAT)* (Rajić et al., 2009; Aminkeng et al., 2015; Aminkeng et al., 2016; Conyers et al., 2018). However, results in relation to these gene variants and anthracycline-induced cardiotoxicity remain inconsistent. One recent study investigated the role of polymorphisms involved in ROS prevention. A study by Krajinovic et al. (2016) found a possible protective effect of *NOS3* (rs1799983) leading to lower risk of developing chronic doxorubicin-induced cardiotoxicity.

#### Other Polymorphisms

Four recent studies discussed polymorphisms in genes not clearly relatable to the known mechanisms of action of anthracyclines.

Wang et al. (2016) found an association between CUGBP Elav-Like Family Member 4 (*CELF4*) (rs1786814) and anthracycline-induced cardiotoxicity. *CELF4* is involved in cardiac dysfunction and fibrosis. In this study, patients carrying *CELF4* (rs1786814) had a higher risk of developing anthracycline- induced cardiotoxicity. This risk increased significantly when patients received an anthracycline dose above 300 mg/m^2^. Hildebrandt et al. (2017) showed a significant protective effect of 1-Phosphatidylinositol-4,5-bisphosphate phosphodiesterase epsilon-1 (*PLCE1*) (rs932764) and ATPase Plasma Membrane Ca2+ Transporting 1 (*ATP2B1)* (rs17249754) in chronic anthracycline-induced cardiotoxicity. The anthracyclines used were not specified in this study. *PLCE1* and *ATP2B1* are involved in calcium signaling in cells. Krajinovic et al. (2016) showed no significant associations between polymorphisms in MutL homolog 1 (*MLH1*)*, MLH2* and *GSTs* and chronic doxorubicin-induced cardiotoxicity. *MLH1* and *MLH2* are genes playing a role in DNA repair and *GSTs* are detoxifying enzymes. Another recent study by Singh et al. (2020) investigated the role of *GSTM1* in chronic anthracycline-induced cardiotoxicity. They found that patients carrying *GSTM1* null genotype (i.e. enzyme activity is absent) had an increased risk of cardiomyopathie. This risk did not increase with patients who received anthracycline-doses of ≥250 mg/m^2^. Ruiz-Pinto et al. (2017) showed a significant association between chronic anthracycline (i.e. doxorubicin, daunorubicin or epirubicin)-induced cardiotoxicity and G protein-coupled receptor family 35 (*GPR35*) (rs12468485) gene. Patients carrying *GRP35* (rs12468485) developed cardiotoxicity more frequently.

#### Other Anthracycline-Induced Toxicities

One recent study investigated the role of Glucose-6-fosfaat-dehydrogenase (*G6PD*) (gene involved in the pathway of detoxifying ROS) normal or deficient enzyme function with daunorubicin-induced hematotoxicity. A retrospective study of Robinson et al. (2019) showed no association between *G6PD* normal of deficient function and daunorubicin-induced hematotoxicity.

Most recent studies focused on polymorphisms in metabolism and transport genes. More recently, other genetic variants are discovered through GWAS which play a possible role in toxicities of anthracyclines. However, there is no consensus on the role of metabolism, transport and other gene variants in anthracycline-induced toxicities, and more research is needed to confirm associated findings and propose dose adjustments to minimalize anthracycline-induced (cardio)toxicity.

### Asparaginase

Asparaginase is a chemotherapeutic agent derived from bacteria *E. Coli* (Oncaspar^®^) and *Erwinia chrysanthemi* (Erwinase^®^) and is used in the treatment for acute lymphatic leukemia (Verma et al., 2007). Asparaginase catalyzes the deamination of asparagine to aspartic acid and ammonia, leading to a reduced serum asparagine concentration and leukemic cell death (Hijiya and van der Sluis, 2016). Unfortunately, asparaginase causes severe toxicities such as hypersensitivity, hepatotoxicity, pancreatitis, and thrombosis. These toxicities lead to therapy resistance, treatment discontinuation and eventually poor clinical outcomes (Hijiya and van der Sluis, 2016; Rank et al., 2019). A study by Rank et al. (2019) showed that pancreatitis occurred in up to 11% of children treated with asparaginase and 44% of patients re-exposed to asparaginase experienced a second episode of pancreatitis.

#### Genetic Variances and Toxicity

Over the last years, an increasing number of studies have reported associations between genetic variants and asparaginase toxicities (Abaji and Krajinovic, 2016; Lee and Yang, 2017; Lopez-Santillan et al., 2017; Rank et al., 2019). Genetic variants in asparagine synthase (*ASNS*), human leukocyte antigens (*HLA*) (Abaji and Krajinovic, 2016) and the glutamate Ionotropic Receptor AMPA Type Subunit 1 (*GRIA1*) have been found to influence asparaginase toxicity (Lee and Yang, 2017; Lopez-Santillan et al., 2017). Recently, eight studies investigated four asparaginase-induced toxicities (hypersensitivity, pancreatitis, thrombosis, and hepatotoxicity).

One GWAS by Abaji et al. (2017) assessed the association between asparaginase-induced hypersensitivity, pancreatitis, and thrombosis and polymorphisms. Three genetic variants, transporter *SLC7A13* (rs9656982), Myb-binding protein 1A *(MYBBP1A)* (rs3809849) (involved in embryonic and cellular development such as mitosis) and YTH Domain Containing 2 (*YTHDC2)* (rs75714066) (involved in regulate mRNA translation and stability), were associated with a higher risk of developing hypersensitivity. Three polymorphisms, ADAM Metallopeptidase With Thrombospondin Type 1 Motif 17 (*ADAMTS17)* (rs72755233) (function unknown), *MYBBP1A* (rs3809849) (involved in many cellular processes such as syntheses of ribosomal DNA) and Sperm Antigen With Calponin Homology And Coiled-Coil Domains 1 (*SPECC1*) (rs9908032) (function unknown), were associated with a higher risk of pancreatitis. Six polymorphisms were associated with a higher risk of thrombosis. These were Polycystin 2 Like 1, Transient Receptor Potential Cation Channel (*PKD2L1*) (rs6584356) (involved in cell-cell/matrix interactions), Ras And Rab Interactor 3 (*RIN3)* (rs3742717) (functions as a guanine nucleotide exchange for genes *RAB5B* and *RAB31*), Sperm Flagellar 2 (*SPEF2)* (rs34708521) (involved in axoneme development), Macrophage Expressed 1 (*MPEG1)* (rs7926933) (involved in cell cycle), interleukin-16 (*IL16)* (rs11556218) (involved in immune system), and *SLC39A12* (rs62619938).

A GWAS by Højfeldt et al. (2019) found a significant higher risk of hypersensitivity with CCR4-NOT Transcription Complex Subunit 3 (*CNOT3)* (rs73062673). Among other functions, this gene is involved in the regulation of human leukocyte antigen (HLA) gene transcription. While no significance was reached on a genome-wide significance level, Højfeldt et al. also discovered two gene risk variants (i.e. *HLA-DQA1* (rs9272131) and the antigen peptide transporter 2 (*TAP*2) which showed a higher frequency of asparaginase hypersensitivity with patients carrying these variants. These results show that variants in the HLA regions as well as genes regulating expression of these variants are involved in asparaginase hypersensitivity. To further investigate the role of HLA gene variants and asparaginase hypersensitivity, a study by Kutszegi et al. (2017) investigated *HLADRB1, HLADQB1*, and *HLADQA1* alleles (both HLA class II alleles). They showed a significant higher risk of developing hypersensitivity for HLA-DRB1*07:01, HLA-DBQ1*02:02, and HLA-DQA1*02:01 carriers. Also, 27 amino acid positions in HLA class II alleles were found to be significant association with a higher risk for hypersensitivity as well as two haplotypes. These findings are replicated and also confirmed by Gagné et al. (2020).

Three GWAS studies analyzing multiple polymorphisms showed significant associations with asparaginase-induced pancreatitis (Liu et al., 2016; Liu et al., 2017; Wolthers et al., 2017). A GWAS by Liu et al. (2016) generated sixteen carboxypeptidase A2 (*CPA2)* single-nucleotide polymorphisms (SNP) that were associated with pancreatitis (highest association with rs199695765). However, this could not be reproduced by Wolthers et al. (2017). Wolthers et al. (2017) found associations with fourteen SNPs in the theunc-51-like kinase 2 (*ULK2*) gene (highest association with rs281366) and one SNP in G-protein signaling 6 (*RGS6*) gene (rs17179470), nuclear factor of activated T cells 2 (*NFATC2*, rs62228256), pancreatic secretory trypsin inhibitor (*SPINK1*, rs17107315), chymotrypsin C (*CTRC*, rs10436957), and Claudin-2 (*CLDN2*, rs4409525) (Wolthers et al., 2019). The proteases cationic and anionic (*PRSS1-PRSS2*, rs13228878, and rs10273639) reduced the risk of pancreatitis (Wolthers et al., 2019).

One GWAS by Liu et al. (2017) investigated the role of polymorphisms in hepatotoxicity during asparaginase treatment. They found that higher alanine aminotransferase (ALT) levels were associated with patatin-like phospholipase domain-containing protein 3 *(PNPLA3)* (rs738409) (involved in the balance of energy usage and storage in adipocytes).

Studies included in this review added new knowledge to genes and polymorphisms that could play a role in asparaginase toxicity. Previous studies found associations between polymorphisms in asparagine synthase (*ASNS* gene), human leukocyte antigens (*HLA* gene) (Abaji and Krajinovic, 2016) and the glutamate receptor (*GRIA1* gene) and asparaginase toxicity (Lee and Yang, 2017; Lopez-Santillan et al., 2017; Hojfeldt et al., 2019).

### Methotrexate

Methotrexate (MTX) is widely used in pediatric oncology treatment protocols of both hematological malignancies (including ALL) as well as solid tumors. MTX is administrated through various ways of administrations and dosages. These include high intravenous (IV) dosage (>500 mg/m^2^, up to 12 g/m^2^), low oral dosages during the maintenance phase as well as intrathecal administration. The drug has shown great benefit in many cancer treatments, but is also associated with various toxicities, ranging from gastro-intestinal toxicity (including severe mucositis), hepatic toxicity, neurotoxicity, nephrotoxicity, and hematological toxicity. These toxicities show large inter-individual differences in pediatric patients. Not only toxicity has been found to be unpredictable, also variations in MTX activity and resistance or reduced sensitivity have been seen in clinical settings.

#### Metabolism and Transport

MTX acts by inhibiting the folate acid cycle, resulting in impairing nucleic acid syntheses. Its pharmacological action follows a complex pattern, with many metabolic enzymes, transporters, and targets. Genetic variants may influence the pharmacological action of MTX in several ways, and many candidate polymorphisms have been studied in relation to folate pathways or MTX metabolism, in search for correlation with response or toxicity of MTX.

#### Genetic Variances and Toxicity

**Figure 3:**
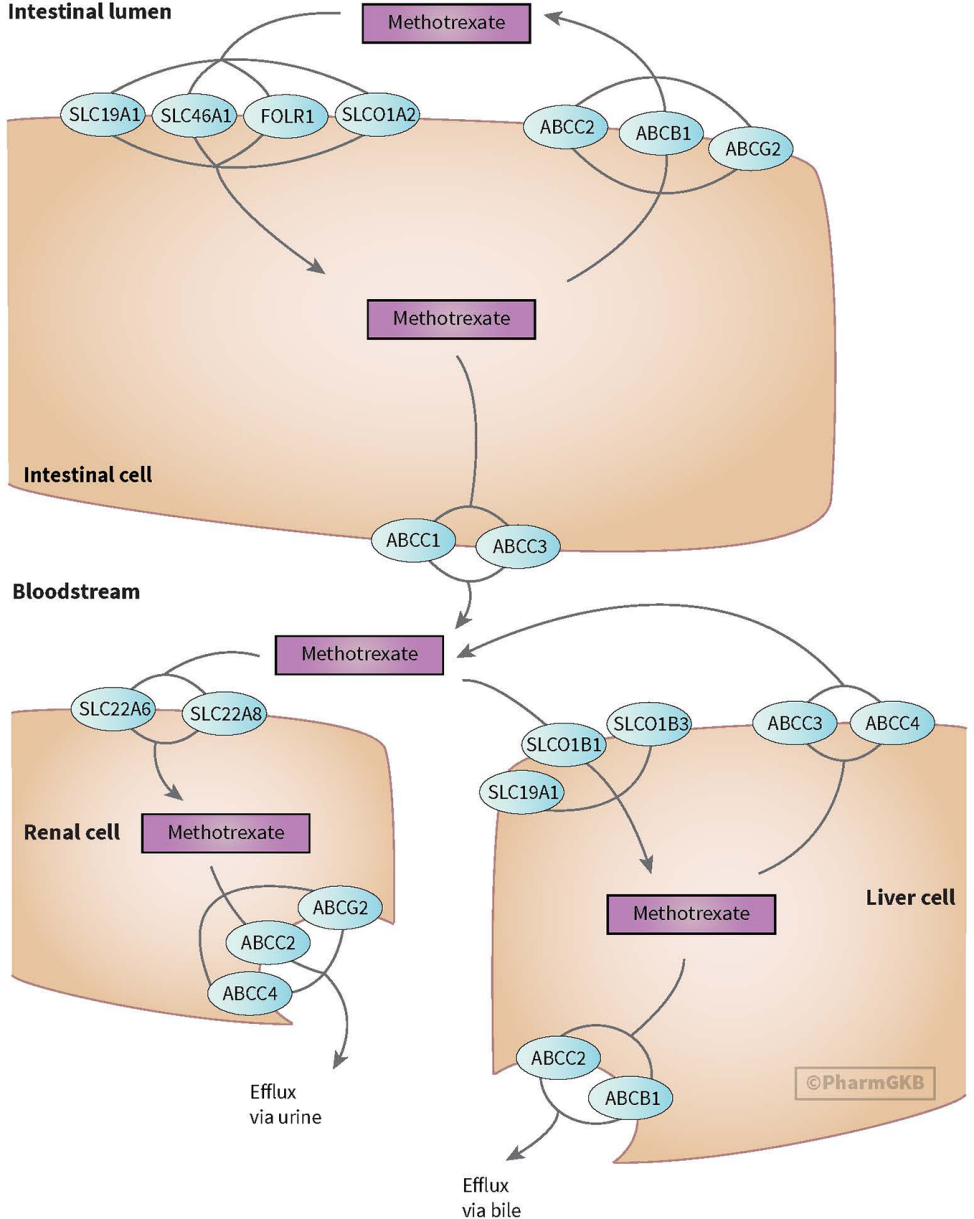
Figure used with permission of PharmGKB (Mikkelsen et al., 2011; Methotrexate Pathway, 2020) Genes involved in transport of methotrexate (Mikkelsen et al., 2011; Methotrexate Pathway, 2020).

MTX enters the cell through active transport through reduced folate carrier (*SLCO1B1/RFC1*). Efflux transporter gene belongs to the ABC superfamily, including ABC transporters such as *ABCB1*. The OATP transporter family is expressed in a variety of tissues and organs important for, among others, MTX elimination. Across the blood-brain barrier, MTX undergoes saturable efflux, presumably through *ABCG2* and organic anion transporter OAT3 (see **Figure 3**) (Mikkelsen et al., 2011; Franca et al., 2017; Methotrexate Pathway, 2020). MTX is intracellularly metabolized to its active polyglutamate form (MTX-PGs) by folylpolyglutamate-synthetase (*FPGS*) and gamma-glutamyl hydrolase (*GGH*) enzymes. GGH enzymes catalyzes the removal of polyglutamates

Polymorphism in genes, coding for these transporters, have been studied for associations with an altered clearance and sensitivity of MTX (e.g. *ABCB1* and *ABCC4* genes), but results have been inconsistent (Ramírez-Pacheco et al., 2016; Hegyi et al., 2017). Genetic polymorphism in the ABC-transporter genes are believed to result in failure of the excretory system, prolonged MTX exposure and have been associated with higher incidence of myelosuppression during MTX treatment (Mlakar et al., 2016).

Recently, *SLCO1B1* gene (encoding for OATP1B1 transporter) has gained interest. The OATP1B1 transporter, located on the membrane of human hepatocytes, mediates disposition of many medications. *SLCO1B1* polymorphisms have been associated with lower MTX clearance, nephrotoxicity, and GI toxicity (Liu et al., 2017). While the association for *SLCO1B1* could not be replicated by Razali et al. (2020), they did show a possible increased risk of leukopenia with *ABCC2* (rs717620) or the transcriptional factor of B lymphocyte progenitors (*ARID5B*, rs4948496) carriers. Patients with genetic variants in *SLCO1B1* may benefit from increased precautionary measures like more aggressive hydration and alkalization (Mlakar et al., 2016).

*SLCO1A2* (encoding for OATP1A2 transporter) plays a role in MTX elimination. A microRNA (miR) binding site polymorphism in *SLCO1A2* (rs4149009) showed delayed MTX excretion in Chinese population (Wang et al., 2018).

Preliminary evidence showed that *SLC19A1* polymorphism (*SLC19A1* 80G>A), an influx transporter involved in MTX uptake in gut and liver cells, may have a protective effect on the occurrence of mucositis (Park and Shin, 2016; Kotnik et al., 2017). *GGH* polymorphism (*GGH_16T/C*) has been described in relation to MTX associated hepatotoxicity in osteosarcoma patients (Hattinger et al., 2016).

Based on recent studies, genetic variance in transporter or metabolizing enzymes is expected to have influence on the exposure of MTX and its toxicity. However, given the complex route of metabolism and transport of MTX, a simple PGx model will probably not suffice.

#### Genetic Variances and Target

Both MTX and MTX-PGs inhibit dihydrofolate reductase (*DHFR*), an enzyme that catalyzes the conversion of dihydrofolate to its active form tetrahydrofolate. Tetrahydrofolate deficiency leads to the depletion of intracellular folates, resulting in decreased synthesis of nucleic acids and cell death. MTX-PGs also interfere with methylenetetrahydrofolate reductase (*MTHFR*), an enzyme which plays a major part in intracellular folate metabolism. Another enzyme target of MTX is thymidylate synthetase (*TYMS*), responsible for synthesis of a precursor of DNA synthesis.

Variants in *MTHFR* activity have been described and the role of *MTHFR* polymorphism (mainly C677T and A1298C genotypes) in relation to toxicity has been studied by several groups (Campbell et al., 2016; Ramírez-Pacheco et al., 2016; Mahmoud et al., 2018; Zhu et al., 2018; Yousef et al., 2019). However, recent reviews summarized the available data and showed ambiguous results (Umerez et al., 2017; Yao et al., 2019), concluding no clear correlation could be established between *MTHFR* polymorphism and MTX toxicity or relapse data.

*DHFR* and *TYMS* genes have also been studied in smaller cohorts in relation to hematological toxicity of MTX and to intrinsic resistance to MTX (Yousef et al., 2019). However, these results have also been inconclusive.

##### Neurotoxicity, Hepatotoxicity, and Mucositis

MTX and MTX-polyglutamates (MTX-PGs) interfere with the adenosine pathway by inhibiting 5-aminoimidazole-4-carboxamide ribonucleotide formiltransferase (*ATIC*) and promoting adenosine release. This pathway has been implicated in MTX-associated neurotoxicity. Adenosine receptor *ADORA2A* polymorphisms have been associated with MTX related leukoencephalopathy in a small cohort study (Tsujimoto et al., 2016) and with an increased risk on hepatotoxicity (Franca et al., 2017). A study performed by Gutierrez-Camino et al. (2017b) replicated results showing an association between MTX-induced mucositis and miR-1206 variant (rs2114358) in ALL patients.

In summary, many efforts have been undertaken to associate polymorphism in enzymes involved in metabolic routes or targets of MTX with toxicity or activity. These were mainly based upon pediatric cohorts of patients with ALL or osteosarcoma. Despite several positive associations, replication in other cohorts has been difficult and evidence for the association between polymorphism and MTX toxicity is still inconsistent. Currently, pharmacokinetic (PK)/pharmacodynamic (PD) monitoring of MTX treatment is still mandatory as the genomic complexity associated with MTX treatment hampers the preemptive use of PGx to predict variability in toxicity or response in individual patients.

### Platinum Compounds

In pediatric oncology, platinum compounds such as cisplatin and carboplatin are widely used in solid malignancy and neuro oncology treatments. Similar to alkylating agents, they cause DNA damage by establishing crosslinks within and between DNA strands. Characteristic toxicities of platinum compounds include nephrotoxicity, neurotoxicity, and ototoxicity.

#### Metabolism and Transport

Platinum compound transport is handled by several enzymes, including copper uptake protein 1 (*CTR1*), *ABCC2*, copper-transporting P-type ATPase (*ATP7A*), and *ATP7B*. Inside the nucleus, platinum-DNA adducts are formed. Several mechanisms influence the impact of DNA damage caused by platinum compounds, including recognition of platinum-DNA adducts (*HMGB1*), DNA mismatch repair (*MSH2, MSH6, MLH1*, and PMS1 Homolog 2, Mismatch Repair System Component (*PMS2*), nucleotide excision repair (X-Ray Repair Cross Complementing 1 (*XRCC1*), ERCC excision repair 1, endonuclease non-catalytic subunit (*ERCC1*), *ERCC2, ERCC3, ERCC4, ERCC6*, DNA Damage Recognition and Repair Factor *(XPA)* and SWItch/Sucrose Non-Fermentable (*SWI/SNF*) and translesion synthesis (DNA Polymerase Eta *(POLH)* and DNA polymerase Beta (*POBL*). Furthermore, several genes (Myeloperoxidase (*MPO*), superoxide dismutase (*SOD1), GSTM1, NQO1, GSTP1, GSTT1, MT1A*, and *MT2A*) may lower the intracellular concentration of platinum compounds (Platinum Pathway Pharmacokinetics/Pharmacodynamics, 2020).

#### Genetic Variances and Toxicity

##### Nephrotoxicity

Platinum compounds, mainly cisplatin, cause damage to the proximal tubules in the kidney, leading to acute kidney injury and electrolyte disturbances. Variants in *ERCC1* (rs3212986) (Khrunin et al., 2010; Tzvetkov et al., 2011), *EPHX1* (rs1051740) (Khrunin et al., 2014), organic cation transporter-2 *(OCT2)* (rs596881) and *CTR1* (rs12686377 and rs7851395) (Chang et al., 2017) have been associated with a reduced risk of renal toxicity in adult patients. All these genes play a role in platinum compound uptake and handling. No recent PGx studies were found regarding platinum compound nephrotoxicity in pediatric oncology populations.

##### Neurotoxicity

Platinum-induced peripheral neuropathy may lead to (irreversible) sensory and motor dysfunction. It is a dose-dependent phenomenon, which could be progressive months after treatment, leading to significant long-term disability. In a cohort of adult testicular cancer survivors, a GWAS revealed a correlation between reduced expression of Regulation Of Nuclear Pre-MRNA Domain Containing 1B *(RPRD1B)* and cisplatin-induced peripheral neuropathy (Dolan et al., 2017). *RPRD1B* is thought to play an important role in several DNA repair mechanisms. There are no data available on PGx of platinum-induced neuropathy in children.

##### Ototoxicity

Among platinum compounds, cisplatin imposes the highest risk of irreversible sensorineural hearing loss. Known concomitant risk factors include higher cumulative dose, younger age, treatment with additional ototoxic drugs and cranial irradiation. Several genetic variants related to cisplatin ototoxicity, includes rs12201199 in *TPMT*, rs9332377 in catechol-O-methyltransferase (*COMT*) (Ross et al., 2009), and rs62283056 in Wolframin ER Transmembrane Glycoprotein (*WFS1*) (Wheeler et al., 2017). However, results concerning these genes are conflicting (Thiesen et al., 2017).

A genetic variant (rs1872328) in Acylphosphatase 2 (*ACYP2*), coding for a protein thought to be responsible for hair cell development, was found to be significantly associated with hearing loss in pediatric patients treated with cisplatin for embryonal tumors (Xu et al., 2015) and osteosarcomas (Vos et al., 2016). Recently, a variant in *GSTP1* (rs1695), coding for a detoxification enzyme, was found to be associated with an elevated risk of hearing loss in pediatric patients treated with cisplatin or carboplatin (Liberman et al., 2018). Another recent study investigated the role of genes responsible for nucleotide excision repair in DNA (Turan et al., 2019). However, no significant association between ototoxicity and *ERCC1* (rs25487), *ERCC2* (rs13181), and *XRCC1* (rs11615) was found.

Apart from genetic factors, epigenetic factors influencing cisplatin ototoxicity have recently come to attention. In a pediatric medulloblastoma and primitive neuroectodermal tumor patient cohort, Brown et al. (2017) found an association between increased cisplatin ototoxicity susceptibility and a methylation locus (cg14010619) in the P21 (RAC1) Activated Kinase 4 (*PAK4* gene). This gene is responsible for stereociliary bundle migration in inner and outer cochlear hair cells.

In conclusion, PGx studies concerning platinum compound toxicity in pediatric populations have so far focused on ototoxicity. The results of these studies, however, have not yet resulted in PGx based dosing recommendations.

### Glucocorticosteroids

Glucocorticosteroids (GCs) play a major role in the treatment of pediatric cancer. Despite significant benefits of high-dose GCs, treatment is associated with toxicities like hepatotoxicity, hypertension, muscle wasting, metabolic effects, neuro-psychiatric effects, osteonecrosis, and osteoporosis.

#### Metabolism and Transport

Glucocorticoids (GC) exert their activity by reducing cell proliferation and promoting apoptosis or cell arrest by binding to intracytoplasmic glucocorticoid receptors (*GR/NR3C1*). Three polymorphisms in the *NR3C1* gene are known to be associated with reduced sensitivity of GCs: *TthIIII* (rs10052957), *ER22/23K* (rs6189/rs6190), and *GR-9β* (rs6198). In contrast, *N363S* (rs6195) and *BC1I* (rs41423247) are associated with an increased sensitivity to GC (van Rossum and Lamberts, 2004).

GCs are metabolized in the liver primarily *via CYP3A4*. Expression of *CYP3A4* varies between individuals and has been associated with outcome in adult cancers (Miyoshi et al., 2002). Differences in expression of *CYP3A4* may be explained by polymorphisms in nuclear receptors (*NR112*) that are involved in the transcriptional regulation of *CYP3A4* (Lamba et al., 2010).

GCs are mainly transported by a multidrug resistance protein encoded by *ABCB1*. Inter-individual variability in the expression of multidrug resistant gene (*MDR1*) is observed and may influence the efficacy of GCs. Furthermore, *GSTs* are involved in several cellular processes and genes of the BCL2 family are involved in the apoptotic response of GCs.

##### Genetic Variances and Toxicity

Numerous studies have investigated the potential link between polymorphisms in the *NR3C1, CYP3A4, ABCB1, GST*, and *BCL2* genes and GC response and toxicity, but have yielded conflicting results and so far, none of the studied genetic variants has been implemented in the treatment of cancer. In the past four years only a few new studies have been published.

A recent study including 346 pediatric ALL patients showed that patients with N363S genotype in the *NR3C1* gene were more prone to steroid-induced toxicities during ALL treatment. Hepatotoxicity was significantly more frequent among patients with N363S genotype than non-carriers (Eipel et al., 2016). This underlines the hypothesis that increased GC sensitivity due to a polymorphism might lead to increased susceptibility to steroid-induced toxicity. A study by ElFayoumi et al. (2018) associated a polymorphism in the *ABCB1* (rs1045642 C3435T) gene with life-threatening infections due to GC treatment.

Bone fractures and osteonecrosis have most frequently been attributed to exposure to GCs. Two polymorphisms in the *BCL2L11* gene (891T>G rs2241843 and 29201C>T rs724710), encoding Bim protein, were significantly associated with steroid-induced osteonecrosis in children with ALL. The 891T>G was also confirmed in a replication cohort and influenced *in vitro* Bim gamma isoform levels. Bim proteins are believed to be involved in the sensitivity of ALL cells to corticosteroid-induced apoptosis (Plesa et al., 2019). A GWAS in children with ALL and osteonecrosis showed that the SNP rs10989692 near the glutamate receptor GRIN3A locus, was associated with osteonecrosis (Karol et al., 2015). Glutamate receptor variants were previously associated with arterial embolism and thrombosis (Lin et al., 2013). GCs have been shown to induce the expression of glutamine synthetase in osteoblasts (Olkku et al., 2004). Hence, variations in the glutamate receptors may contribute to vascular events leading to osteonecrosis in patients exposed to GC therapy (Karol et al., 2015). Meanwhile bone toxicity has mainly been considered a consequence of exposure to corticosteroids during ALL therapy (Mattano et al., 2000; Girard et al., 2013). Recently, the TS variant 2R/2R was associated with increased rise of osteonecrosis among children younger than 10 years at diagnosis suggesting that MTX may play a pathophysiologic role in the development of osteonecrosis (Finkelstein et al., 2017). Although evidence is limited, published data describe a positive association between polymorphisms in GC pathways and the efficacy and toxicity of GCs. Its impact on outcome is debatable since resistance to GC might be overcome by the effect of combination drug therapy. Concerning GC-induced toxicity larger studies are needed to investigate the role of genetic polymorphisms in the development of GC-induced toxicity to avoid severe complications.

### Thiopurines

6-Mercaptopurine (6MP) is the cornerstone of the maintenance phase of ALL treatment in children and is used more often than its analogue tioguanine (TG). 6MP is required continuously for 2 to 3 years in leukemia treatment and used in oral dosages varying between 25 and 75 mg/m^2^. Known for its narrow therapeutic window, 6MP is able to cause severe toxicities including myelosuppression, hepatotoxicity, and GI toxicity. 6MP treatment interruption is known to increase the risk of relapse. It is therefore of great importance to find and maintain the optimal 6MP dosage in ALL patients.

#### Metabolism and Transport

6MP is converted intracellularly by hypoxanthine guanine phosphoribosyl transferase (HPRT) into active 6-thioguanine nucleotides (6TG) which are incorporated into DNA, causing cell death. 6MP is methylated by the enzyme thiopurine S-methyltransferase (TPMT) into 6-methylmercapturine (6-MMP). Methylated 6MP metabolites also contribute to the cytotoxic effects of 6MP by inhibiting *de novo* synthesis of purines. Increased levels of 6-TGN and 6-MMP have been associated with an increased risk of toxicity (Koutsilieri et al., 2019).

TPMP activity is well studied and shown to be highly variable among individuals, although the incidence of genetic variants differs between ethnic populations (Jimenez-Morales et al., 2016). In Caucasians, 90% to 95% of subjects have a normal/high TPMT activity, 5% to 10% reduced and around 0.5% an absent enzymatic activity (Franca et al., 2019). In Asian and Hispanic population, the incidence of variant TPMT genes is much lower (Koutsilieri et al., 2019).

Three variant alleles TPMT*2 (G238C), TPMT*3A (G460A and A719G) and TPMT*3C (A719G), account for more than 95% of the inherited variability in TPMT enzyme activity (Conyers et al., 2018), although more TPMT deficient variants have recently been identified, with a less frequent occurrence (Koutsilieri et al., 2019).

#### Genetic Variances and Toxicity

Polymorphisms in TPMT are well studied in relation to the risk of toxicity (mostly severe myelosuppression) and corresponding dosage adjustments (Jimenez-Morales et al., 2016). This has lead to implementation of Federal Drugs Authority (FDA) and European Medical Agency (EMA) supporting clinical guidelines for preemptive testing of TPMT, corresponding with lower starting dosages of 6MP for poor and intermediate metabolizers (Relling et al., 2020). In this review, one study examining the tolerable dose and treatment outcome within patients carrying *TPMT* and nudix hydrolase 15 (*NUDT15*) genetic variants is included, while dose adjustments for *TPMT* and *NUDT15* intermediate and/or poor metabolizers are already used in practice (Liang et al., 2016).

The discovery of frequent thiopurine-induced myelosuppression in Asian populations, while *TPMT* variants rarely occur in these ethnic populations, has led to several studies on the role of *NUDT15* and *ITPA* gene variants (Liang et al., 2016; Milosevic et al., 2018). *NUDT15* dephosphorylates thiopurine active metabolites and reduces the formation of active 6TG nucleotides, whereas *ITPA* is assumed to stimulate the formation of 6TG nucleotides. Results with respect to the influence of *ITPA* polymorphism in relation to myelosuppression, hepatotoxicity or TGN formation have not been consistent and its clinical relevance is still controversial (Gerbek et al., 2018; Milosevic et al., 2018; Soler et al., 2018; Zhou et al., 2018; Khera et al., 2019; Wahlund et al., 2020). In contrast, in many recent studies, *NUDT15* gene defects (rs116855232, rs186364861, rs869320766, rs201094029) have shown to result in increased TG availability for incorporation into DNA and have consistently been associated with TG toxicity including early myelosuppression (Chiengthong et al., 2016; Moriyama et al., 2016; Lee and Yang, 2017; Moriyama et al., 2017; Moriyama et al., 2017; Soler et al., 2018; Choi et al., 2019; Khera et al., 2019; Koutsilieri et al., 2019). The incidence of *NUDT15* gene defects is higher in Asian and Hispanic populations, but *NUDT15* genetic polymorphism has also been described in European patients (Schaeffeler et al., 2019). Therefore, the most recent CPIC guideline on thiopurines recommends both TPMT and NUDT genotyping (Koutsilieri et al., 2019; Relling et al., 2019).

Other SNPs that have been studied in relation to thiopurine sensitivity are variants within the phosphoribosylglycinamide formyltransferase *(GART)* gene (involved in folate cycle), Molybdenum Cofactor Sulfurase *(MOCOS)* gene (involved in thiopurine metabolism) and Protein Kinase C And Casein Kinase Substrate In Neurons 2 *(PACSIN2)* gene (involved in thiopurine metabolism (Smid et al., 2016; Franca et al., 2019). Polymorphisms in *PACSIN2* have been associated with an increased risk of GI and hematologic toxicity during 6MP treatment. However, results have not been as convincing as *TPMT* and *NUDT15* polymorphism (Choi et al., 2019). Genetic variants in Apurinic/Apyrimidinic Endodeoxyribonuclease 1 *(APEX1)* have been studied in relation to a more general sensitivity to antimetabolite active drugs, as human *APEX1* is involved in DNA base excision repair pathway. In Asian populations *APEX1* (rs2307486) variant resulted in an increased risk of 6MP-related early onset neutropenia (Kim et al., 2018).

Transporter genes have also been studied and variants in the *ABCB1* gene (C1236T) have found to be associated with increased sensitivity to 6MP (Gervasini et al., 2017), as well as variant genes in efflux transporter *ABCC4* (Kim et al., 2018; Tanaka et al., 2018). These findings have been reported in small studies and need confirmation before being introduced in clinical practice.

In conclusion, in recent years evidence for preemptive TPMT and NUDT15 genotyping in facilitating tailored thiopurine treatment has been firmly established.

### Topoisomerase Inhibitors

#### Topoisomerase I Inhibitors

Topoisomerase I (Top 1) inhibitors act by preventing ligation of single strand breaks in DNA. In pediatric oncology, topoisomerase I inhibitors (such as irinotecan and topotecan) are used in the treatment of multiple solid malignancies. Topoisomerase I inhibitors are known to cause serious toxicities, such as myelosuppression and severe diarrhea. As not much is known on pharmacogenetic mechanisms involved in topotecan metabolism, this section will focus on irinotecan.

##### Metabolism and Transport of Top 1 Inhibitors

Irinotecan is a prodrug that is transported over hepatocyte cell membranes by several transporters (*SLCO1B1, ABCB1, ABCC1, ABCC2*, and *ABCG2*). In the cytosol, it is converted to active compound SN-38 *via* carboxylesterase (*CES1*) and *CES2*. SN-38 actively inhibits topoisomerase I, forcing cells to arrest in S phase and leading to cell death. UGT1A1 detoxifies SN-38 through glucuronidation. UGT1A1 converts SN-38 into soluble and non-toxic SN-38 glucuronic acid, which is released into the intestines. As diarrhea is caused by accumulation of SN-38 in intestinal mucosa, detoxification to SN-38 glucuronic acid is important for limiting this toxicity. Furthermore, irinotecan itself can be converted to inactive metabolites APC and NPC by *CYP3A4* and *CYP3A5*. NPC can be converted to SN-38 by *CES1* and *CES2* (Mlakar et al., 2016).

##### Genetic Variances and Toxicity in Top 1 Inhibitors

Two genetic variants in *UGT1A1* are known to significantly influence irinotecan metabolism. UGT1A1*28 allele (rs8175347) is more prevalent in Caucasian and African American populations compared to East Asian populations (Zhang et al., 2007). It is a thymidine-adenosine (TA) repeat in the *UGT1A1* promoter region, impairing transcription and therefore UGT1A1 enzyme function and SN-38 detoxification. This variant is associated with severe neutropenia and late diarrhea. According to the FDA drug label for irinotecan, dose reduction is recommended in adult UGT1A1*28 poor metabolizers. However, evidence in a pediatric population showed no severe toxicity when a low dose of irinotecan was used (Stewart et al., 2007). UGT1A1*6 allele (rs4148323) mainly occurs in East Asian populations. It is characterized by G71 to R substitution and causes decreased UGT1A1 enzyme function similar to the UGT1A1*28 allele (Etienne-Grimaldi et al., 2015).

Efforts to identify genetic variants that influence irinotecan toxicity are ongoing. Trubicka et al. (2017) found that pediatric medulloblastoma patients with variants in DNA repair genes (such as *MSH2*, *RAD50*, nibrin (*NBN*), Fanconi anemia complementation group (*FANCM*), and exonuclease 1 (*EXO1*)) experienced significantly more adverse effects from treatment containing irinotecan. However, as these patients were treated with multiple drugs concomitantly, functional studies will be needed to elucidate the underlying mechanisms.

#### Topoisomerase II Inhibitors

Topoisomerase II (Top 2) inhibitors, such as etoposide, prevent ligation of double strand breaks in DNA. Etoposide is widely used in the treatment of both solid and hematological malignancies in pediatric oncology. Toxicities include myelotoxicity and increased long-term risk of secondary malignancies (such as myeloid leukemia).

Although many enzymes are known to play a role in metabolism and transport of Top 2 inhibitors (among which are CYP3A4, CYP 3A5, and UGT1A), no variants of significant clinical impact have been identified to date (Khera et al., 2019).

### Vinca Alkaloids

Vinca alkaloids are included in chemotherapy regimens of hematologic malignancies, solid tumors, and neuro-oncology. Their main mechanism lies in the disruption of microtubule function during cell division, leading to a metaphase arrest in the cell cycle and apoptosis (Moudi et al., 2013; van de Velde et al., 2017; Vinka Alkaloid Pathway, 2020). Peripheral neurotoxicity is a well-known side effect of vinca alkaloids, while primarily of vincristine (VCR). VCR-induced peripheral neuropathy has an incidence rate between 78% and 100% (Kandula et al., 2016) leading to muscle weakness and pain in hand and feet (van de Velde et al., 2017). It is dose-dependent and develops most severe at doses above 2 mg/m^2^ (Park et al., 2013). Therefore, in pediatric oncology a dose maximum of vincristine is fixed at 2 mg to prevent severe neurotoxicity (Kandula et al., 2016).

Genetic polymorphisms are believed to play a role in a patients’ sensitivity for VCR-induced neurotoxicity. These include genes involved in metabolism and transport out of (hepatic) cells of vinca alkaloids as well as genes involved in pharmacodynamics, stabilization, and formation of microtubules and nerves and inherited neuropathy genes (van de Velde et al., 2017). In this review we were only able to update vincristine (VCR) PGx in ALL patients as our literature search revealed no recent studies regarding vinblastine or vinorelbine or other pediatric cancer populations.

#### Metabolism

*CYP3A4* and *CYP3A5* enzymes are involved in the metabolism of vinca alkaloids in the liver and particularly known for VCR metabolism (Vinka Alkaloid Pathway, 2020). Four recent studies showed no association between vincristine (neuro)toxicity and polymorphisms in metabolizing enzymes.

In a retrospective study by Franca et al. (2017) 28 single nucleotide polymorphisms (SNPs) and two deletions genes involved in efficacy and adverse effects were collected to find an association between the polymorphisms and grade III/IV gastrointestinal, hepatic, and neural toxicity. Before adjusting for multiple variables, four genes were found to be associated with vincristine related toxicities. *ITPA* (rs1127354) increased the risk of neurotoxicity and gastrointestinal toxicity, while *ADORA2A* (rs2236624) was found the associated with hepatic toxicity. After adjustment for multiple variables, none of the associations remained significant (Franca et al., 2017). McClain et al. (2016) performed a retrospective study with 239 Hispanic ALL patients, analyzing *CYP3A5* extensive (*1/*1), *CYP3A5* intermediate (*1/*3, *1/*6, *1/*7) and *CYP3A5* poor metabolizers (*3/*3, *3/*6, *3/*7). No significant association was found between *CYP3A5* polymorphisms and vincristine-induced peripheral neuropathy (VIPN). Skiles et al. (2018), included a cohort of 78 Kenyan children to find an association between *CYP3A5* and VIPN. Ninety one percent of the children were *CYP3A5* high-expresser genotypes and none developed neuropathy, leading to no conclusive results.

Results in the past are conflicting, showing associations as well as no associations between *CYP3A4*, CYP3A4*1B, CYP3A5, CYP3A5*3, and VIPN (Kandula et al., 2016; Mora et al., 2016; van de Velde et al., 2017; Conyers et al., 2018).

#### Transport

Vinca alkaloids are transported out of cells through *ABCB1, ABCC1, ABCC2, ABCC3, ABCC10*, and RalA-binding protein 1 (*RALBP1*). Seven recent studies investigated polymorphisms in transport genes and the risk on developing vincristine neurotoxicity. One study showed a significant association between *ABCC1* (rs3784867) and VIPN.

As stated above, Franca et al. (2017) investigated 28 SNPs and two deletions, including *ABCC1* (rs35592, rs246240, rs3784864, rs11075291) and *ABCC2* (rs17222723). Before adjustment for multiple variables, *ABCC1* (rs246240) was associated with increased the risk of neurotoxicity. After adjustment, this result did not reach significance. Lopez-Lopez et al. (2016) performed a retrospective study including 152 B-cell ALL Spanish patients. In this study 150 genetic variants involved in VCR pharmacokinetics and 13 microRNAs were analyzed. A significant higher risk of developing neurotoxicity grades 1 to 4 during vincristine treatment was found for rs3740066 and rs12826 in the *ABCC2* gene. Zgheib et al. (2018) assessed the association between grade II or higher VIPN and *ABCB1* (rs1045642), *ABCB1* (rs1128503) and *ABCC2* (rs717620). The study included 133 Arab ALL patients, where 19.5% developed VIPN. None of the polymorphisms showed a significant association with VIPN. In a retrospective study by Wright et al. (2019), a higher risk of VIPN was associated with *ABCC1* (rs3784867).

Gutierrez-Camino et al. (2017a) performed a retrospective study including 179 Spanish children with B-cell ALL. In this study, the authors analyzed 154 microRNAs (miRNAs) to find an association between the miRNAs and vincristine neurotoxicity. Three miRNAs were found most significant before corrections. These were miR-3117 (rs12402181) involved in *ABCC1* and RalA-binding protein 1 (*RALPBP1*) expression, miR-4481 (rs7896283), and miR-6067 (rs35650931). miR-3317 and miR-6067 were associated with a decrease risk of neurotoxicity during vincristine treatment, while miR-4481 was associated with a higher risk of neurotoxicity. After multivariable correction, none of the miRNAs produced a significant result.

#### Pharmacodynamics, Stability of Microtubules and Neurotoxicity Sensitivity

Polymorphisms in pharmacodynamics (i.e. actin gamma 1 (*ACTG1*) (Ceppi et al., 2014), formation and stabilization of microtubules (i.e. Centrosomal Protein 72) (*CEP72*) (Diouf et al., 2015), Microtubule Associated Protein 4 (*MAP4*) (Ceppi et al., 2014), Capping Actin Protein, Gelsolin Like (*CAPG*) (Ceppi et al., 2014) Tubulin Beta 1 Class VI (*TUBB1*)*, TUBB2A, TUBB2B, TUBB3, TUBB4*) (Ceppi et al., 2014) and genes known to influence neurotoxicity sensitivity (i.e. Charcot-Marie-Tooth disease confirmed in adults) have in the past been associated with vincristine toxicology (Kandula et al., 2016; Lee and Yang, 2017). A special attention is drawn to the *CEP72* gene. In a past preliminary study with ALL children, genetic variants in the promotor region of *CEP72* were associated with a higher risk of developing VIPN (Diouf et al., 2015). This investigation has recently been replicated in more studies. Seven recent studies investigated genetic variations in pharmacodynamics, microtubules stability, and neurotoxicity sensitivity. Two studies found an association with VIPN.

Gutierrez-Camino (2016) showed no significant association between VIPN and *CEP72* (rs924607) in a retrospective Spanish cohort with 142 B-cell ALL patients. McClain et al. (2016) also found no significance with polymorphisms in the *CEP72* gene, while Wright et al. (2019) showed that *CEP72* (rs924607), *SLC547* (rs1013940) (choline transporter), and Alpha Tocopherol Transfer Protein (*TTPA*, binding to vitamin E) (rs1050436) were significant associated with a higher risk of developing neuropathy. No association was found by Zgheib et al. (2018) between VIPN and *CEP72* (rs924607), Ewing’s tumor-associated antigen 1 *(ETAA1*) (rs17032980) and Melatonin Receptor 1B *(MTNR1B)* (rs12786200).

Li et al. (2019) investigated two independent cohorts and used a meta-analysis to assess the association between multiple SNPs and VIPN. One SNP, rs1045644 (encoding for protein cochlin, which is associated with progressive hearing loss and vestibular imbalance), showed a protective effect against neuropathy, while rs7963521 (gene involved in angiogenesis) was associated with a higher risk of VIPN. Martin-Guerrero et al. (2019) used a retrospective cohort with 152 B-cell ALL patients to analyze 24 polymorphisms (in *TUBB1, TUBB2A, TUBB2B, TUBB3, TUBB4, MAPT*, *MIR146a*, *MIR202*, and *MIR411*). Before adjusting for false discovery rate, several gene variants have been associated with vincristine-induced neurotoxicity. Patients carrying *MAPT* (rs11867549), Mir-202 (rs12355840), and *TUBB3* (rs4395073) had a higher risk of developing neurotoxicity. Also several haplotypes were associated with neurotoxicity.

Abaji et al. (2018) screened retrospectively WES data of ALL patients to find possible new gene variants which could be associated with VIPN. In this study, three new gene variants are association with VIPN: Spectrin Repeat Containing Nuclear Envelope Protein 2 (*SYNE2*) (rs2781377), Mitochondrial Ribosomal Protein L47 (*MRPL47*) (rs10513762) and Bromo Adjacent Homology Domain Containing 1 (*BAHD1*) (rs3803357). While *SYNE2* (rs2781377) and *MRPL47* (rs10513762) were associated with a higher risk of developing VIPN, *BAHD1* (rs3803357) showed a protective effect. *SYNE2* is a protein involved in cellular cytoskeletons, DNA damage repair and other cellular processes. *MRPL47* plays a role in the mitochondrial protein synthesis. *BAHD1* is a protein involved in gene silencing

Studies investigated polymorphisms in metabolism, transport, pharmacodynamics, and sensitivity genes of patients to VIPN shows conflicting results. While CEP72 has drawn more attention, its role has yet to be confirmed. The new gene variants associated with VIPN showed by Abaji et al. needs to be replicated by other studies to include these gene variants as risk factors for developing VIPN.

## Discussion

Chemotherapeutics are known for their narrow therapeutic window. While it is of utmost importance to use the right dose to sustain a favorable outcome in pediatric oncology, toxic doses cause poor outcomes, and a worse quality of life for pediatric cancer survivors. Based upon inter-individual differences in occurrence and severity of toxicity between pediatric cancer patients, the need for personalized treatment is apparent. PGx has been introduced to understand and facilitate individual treatments in pediatric oncology.

With this review, we present new developments over the past years concerning PGx within pediatric oncology. We included relevant literature from 2016 onward, revealing new genetic variations as well as new evidence for already known associated genetic variations with chemotherapeutics’ toxicity.

Most studies have focused on genetic variations within metabolism or transport genes. Surprisingly, while CYP enzymes influence drug exposure significantly within other drug categories [e.g. selective serotonin reuptake inhibitors (Relling et al., 2020)], associations with toxicities of alkylating agents, anthracyclines, topoisomerase inhibitors and vinca alkaloids were not consistently found in studies included in this review. ABC transporters play a role in the transport of anthracyclines, methotrexate, platinum compounds, glucocorticosteroids, thiopurines, topoisomerase inhibitors, and vinca alkaloids. SLC transporters are thought to play a role in the transport of anthracyclines, asparaginase, methotrexate, topoisomerase inhibitors, and vinca alkaloids. However, there is slight evidence for robust and reproducible correlations between transporter genes and efficacy or toxicity of chemotherapeutics.

The role of ontogeny in the activity of metabolic enzymes or transporters may play a part when studying patient populations of different age groups, especially in neonates and infants (Mlakar et al., 2016). In our review, we focused on pediatric populations but found no indication that (young) age was considered an independent factor that influences the role of PGx in toxicity of chemotherapeutic drugs. However, neonates and infants are underrepresented in pediatric oncology studies, because of limited occurrence of malignancies in these age groups. Strong evidence for associations between genetic variations in *NUDT15* and *TPMT* and 6MP toxicity have been confirmed in our review and preemptive genetic testing for NUDT15 and *TPMT* variants should be implemented in standard clinical care. Future attention should be focused on standardizing corresponding dosing guidelines and exploring the relevance of less frequent occurring variants of *TPMT* and *NUDT15*. Evidence has been established for the role of *UGT1A1* polymorphisms in irinotecan toxicity in adults, but this still need confirmation in pediatric populations.

Due to improvements in genomic sequencing technologies, research has shifted to genetic variations in pharmacodynamics and cytostatic targets as well as less apparent gene polymorphisms as an explanation for efficacy or toxicity differences of chemotherapeutics in pediatric cancer patients. Variations in genes coding for cytostatic targets (e.g. microtubule stabilization by VCR, glucocorticoid sensitivity, folate activity by MTX) have been included in this review and showed probable contribution to individual differences in response and toxicity. However, there were no decisive conclusions that could be drawn from these studies and recommendations on dose adjustments are not yet established.

We did not include studies on immunotherapy or supportive care drugs. The use of immunotherapy in pediatric oncology has increased in the past years, showing promising results. However, as with chemotherapeutics, individual differences in response and toxicity of immunotherapy within children are reported. In future years, the role of genetics will need to be further elucidated. Supportive care drugs are also of great importance in limiting chemotherapeutics’ toxicities (e.g. neuropathy, diarrhea, infections due to neutropenia). Therefore, optimalization of supportive care dosages is needed and PGx may be beneficial in assessing the correct dosages. Currently, adult guidelines are used to assess the impact of PGx in dosing supportive care drugs in pediatric oncology.

Our review shows that pharmacogenomics in pediatric oncology is still facing challenges. The main problem is inconsistency in results, caused by small population sizes, differences in (statistical) interpretation, variations in sequencing technologies as well as in differences in definition of clinical outcomes. Lack of information on how to use genetic test results to adjust the use or dose of chemotherapeutics hinders broad introduction in clinical practice (Relling et al., 2020). Also, pediatric cancer patients receive combinations of chemotherapeutics, making it difficult to discern the impact of individual genetic variances. Finally, most studies are performed in pediatric ALL patients, leading to limited data on other pediatric cancer types.

In conclusion, pharmacogenomics of chemotherapeutics is complex with multiple genes involved in the process of metabolism, transport and its target mechanisms. Future studies should focus on establishing comprehensive models, integrating pharmacogenomics with pharmacokinetics and pharmacodynamics data to aid dosing guidelines. Standardization of clinical outcome data and toxicity definitions within electronic health records combined with the increased availability of genomic sequences techniques in clinical practice will help to validate these models in larger populations.

## Author Contributions

EB developed the search codes (with libraries from Utrecht University Library) for the databases and collected the data. All authors reviewed the results and contributed to the manuscript. All authors contributed to the article and approved the submitted version.

## Conflict of Interest

The authors declare that the research was conducted in the absence of any commercial or financial relationships that could be construed as a potential conflict of interest.
