# Supplementary material for: Pharmacogenomics as a Tool to Limit Acute and Long-Term Adverse Effects of Chemotherapeutics: An Update in Pediatric Oncology
Source: Front Pharmacol. 2020 Aug 5;11:1184. doi: 10.3389/fphar.2020.01184 (PMC7421781; doi:10.3389/fphar.2020.01184)
Supplement: Supplementary file 1 [file Table_1.pdf]

Table 1: Overview of recent studies (from 2016 onward) analyzing the association between genetic variants and toxicities (and/or other variables)

Chemotherapy agent	*Study design*	*Number of enrolled pediatric patients*	*Diagnosis*	*Toxicitiy and/or other variable*	*Tested genes*	*Gene variant(s)*	*Associated with*	*Odds ratio (95% CI), significance yes/no (p-value)*	*Ref.*
**ALKYLATING AGENTS**									
Cyclophosphamide	Prospective study	49	B-cell Non Hodgkin Lymphoma	Clearance	*CYP2B6, CYP2C19 GSTP1*	*CYP2B6*6*	Lower clearance	Yes (study dose 1 $p = 0.033$ and study dose 5 $p = 0.0028$)	(Veal, Cole et al. 2016)
						*CYP2C19*2* and *CYP2C19*17*	Unknown	No ($p > 0.05$)	
						GSTP1	NA	NA	
Ifosfamide	Case report	3	Osteosarcoma	Encephalopathy	*CYP2B6*	*CYP2B6* (rs4803419), *CYP2B6*6*	Possible increased risk of encephalopathy	NA	(Duflot, Marie-Cardine et al. 2018)
Busulfan	Prospective study	20	Mixed	Pharmacokinetics	*GSTA1 GSTM1 GSTT1*	*GSTA1*A*A, /*B*B/*A*B/ B*A*	Unknown	No ($p > 0.05$)	(Nishikawa, Yamaguchi et al. 2019)
						GSTM1 and *GSTT1* positive or negative	Unknown	No ($p > 0.05$)	
Busulfan	Retrospective population pharmacokinetics (PopPK) study	112	Mixed	Pharmacokinetics	*GSTA1*	*GSTA1*A2*A2/*A2*A3/*A3*A3/*A3/*A2*[#]	7% higher clearance	NA	(Nava, Kassir et al. 2018)
						*GSTA1*B1b/*B1b*[#]	12% lower clearance	NA	
**ANTHRACYCLINES**									
Daunorubicin	Retrospective study	36	Acute Lymphoblastic Leukemia	Pharmacokinetics and cardiotoxicity	*CYP3A5*	*CYP3A5*1*1/*1*3/*3*3*	Unknown	No ($p > 0.05$)	(Huang, Wang et al. 2017)

Table 1: Overview of recent studies (from 2016 onward) analyzing the association between genetic variants and toxicities (and/or other variables)

Doxorubicin, daunorubicin, epirubicin	GWAS Retrospective case control study	93	Mixed	Cardiotoxicity	NA	*GPR35* (rs12468485) CT allele	More frequent chronic cardiotoxicity (anthracycline dose 155 mg/m^2)	NA, Yes (after FDR correction p = 0.03)	(Ruiz-Pinto, Pita et al. 2017)
						ABCB1 polymorphisms	Unknown	No (p > 0.05)	
						SLC28A3 polymorphisms	Unknown	No (p > 0.05)	
Doxorubicin	Retrospective study	251	Acute Lymphoblastic Leukemia	Cardiotoxicity	*ABCC1* *ABCC2* *ABCC5* *ABCB1* *NFKB1* *NQO1* *NOS3* *MLH1* *MLH2* *GSTs*	*ABCC5* (rs7627754) TT genotype	Reduction of ejection fraction and shortening fraction	Yes ($p_{ejectionfraction}$ < 0.0005, $p_{shorteningfraction}$ = 0.001)	(Krajinovic, Elbared et al. 2016)
						NOS3 (rs1799983) TT genotype	Higher ejection fraction	Yes (p = 0.02)	
Daunorubicin	Prospective study	726	Acute Lymphoblastic Leukemia	Hematotoxicity	*G6PD*	*G6PD* normal or deficient	Unknown	No (p > 0.05)	(Robinson, Yang et al. 2019)
Anthracyclines (not specified)	GWAS Follow-up/case control study with cancer survivors	108	Mixed	Cardiotoxicity	NA	*PLCE1* (rs932764)	Decreased risk of cardiotoxicity	OR 0.48 (0.27–0.85) , Yes (p = 0.0068)	(Hildebrandt, t et al. 2017)
						ATP2B1 (rs17249754)	Decreased risk of cardiotoxicity	OR 0.26 (0.07–0.96), Yes (p = 0.040)	
Anthracyclines (not specified)	GWAS Retrospective case control study	430	Mixed	Cardiotoxicity	NA	*CELF4* (rs1786814) GG genotype	Increased risk of cardiotoxicity	OR 2.26 (1.2-4.0), Yes (p = 0.006)	(Wang, Sun et al. 2016)
						CELF4 (rs1786814) GG genotype	Increased risk of cardiotoxicity (anthracycline dose >300 mg/m^2)	OR 10.16 (3.8-27.3), Yes (p > 0.001)	
Anthracyclines (not specified)	Retrospective case-control study	167	Mixed	Cardiotoxicity	*GSTM1*	*GSTM1* null genotype	Increased risk of cardiomyopathy	OR 2.5 (1.2-5.2), Yes (p = 0.01)	(Singh, Wang

Table 1: Overview of recent studies (from 2016 onward) analyzing the association between genetic variants and toxicities (and/or other variables)

	with cancer survivors					*GSTM1* null genotype	Increased risk of cardiomyopathy (anthracycline dose ≥250 mg/m^2)	OR 2.6 (1.15-6.1), Yes (p = 0.02)	et al. 2020)
**ASPARAGINASE**									
Asparaginase	GWAS Retrospective study	302	Acute Lymphoblastic Leukemia	Hypersensitivity, pancreatitis and thrombosis	NA	*SLC7A13* (rs9656982) AG/GG genotype	Increased risk of hypersensitivity	OR 2.1 (1.1-3.9), Yes (p = 0.02)	(Abaji, Gagne et al. 2017)
						MYBBP1A (rs3809849) GC/CC genotype	Increased risk of hypersensitivity	OR 2.4 (1.4-3.9), Yes (p = 0.0006)	
						YTHDC2 (rs75714066) GC/CC genotype	Increased risk of hypersensitivity	OR 3.1 (1.4-7.0), Yes (p = 0.008)	
						ADAMTS17 (rs72755233) GA/AA genotype	Increased risk of pancreatitis	OR 5.6 (1.9-16.3), Yes (p = 0.002)	
						MYBBP1A (rs3809849) GC/CC genotype	Increased risk of pancreatitis	OR 6.9 (1.9-25.2), Yes (p = 0.002)	
						SPECC1 (rs9908032) CG/GG genotype	Increased risk of pancreatitis	OR 3.9 (1.6-9.2), Yes (p = 0.0008)	
						PKD2L1 (rs6584356) CA/AA genotype	Increased risk of thrombosis	OR 5.0 (1.2-20.7), Yes (p = 0.05)	
						RIN3 (rs3742717) CT/TT genotype	Increased risk of thrombosis	OR 13.8 (2.3-82.5), Yes (p = 0.02)	
						SPEF2 (rs34708521) GA/AA genotype	Increased risk of thrombosis	OR 6.1 (1.4-26.9), Yes (p = 0.03)	
						MPEG1 (rs7926933) GA/AA genotype	Increased risk of thrombosis	OR 5.7 (1.5-22.1), Yes (p = 0.01)	
						IL16 (rs11556218) TG/GG genotype	Increased risk of thrombosis	OR 7.4 (1.8-31.2), Yes (p = 0.01)	
						SLC39A12 (rs62619938) CT/TT genotype	Increased risk of thrombosis	4.4 (1.6-11.7), Yes (p = 0.0005)	

Table 1: Overview of recent studies (from 2016 onward) analyzing the association between genetic variants and toxicities (and/or other variables)

Asparaginase	GWAS Retrospective case control study	1494	Acute Lymphoblastic Leukemia	Hypersensitivity	NA	*CNOT3* (rs73062673) C allele	Increased risk of hypersensitivity	OR 3.7 (2.33-5.98), Yes (p = 4.68x10^{-8})	(Hojfeldt, Wolthers et al. 2019)
						Genes located on *HLA-DQA1* and *HLA-DQA2* regions, *TAP2* located on *HLA-DOB* region	Unknown	No (p > 0.05)	
Asparaginase	Retrospective study	359	Acute Lymphoblastic Leukemia	Hypersensitivity	*HLA-DRB1* *HLA-DQB1* *HLA-DQA1*	*HLA-DRB1*07:01* carrier	Higher risk of hypersensitivity	OR 2.86 (1.73-4.75), Yes (p = 4.56x10^{-5})	(Kutszegi, Yang et al. 2017)
						*HLA-DQB1*02:02* carrier	Higher risk of hypersensitivity	OR 2.99 (1.68-5.31), Yes (p = 1.85x10^{-4})	
						*HLA-DQA1*02:01* carrier	Higher risk of hypersensitivity	OR 3.69 (1.99-6.84), Yes (p = 3.45x10^{-5})	
						27 polymorphic amino acid positions in HLA class II alleles, strongest association with position 78 in *HLA-DRB1*	Higher risk of hypersensitivity	OR 4.01 (2.18-7.37), Yes (p = 8.16x10^{-6})	
						*HLA-DRB1*07:01 - HLA-DQB1*02:02* haplotype	Higher risk of hypersensitivity	OR 2.99 (1.68-5.31), Yes (p = 1.85x10^{-4})	
						*HLA-DRB1*07:01 - HLA-DQA1*02:01 - HLA-DQB1*02:02* haplotype	Increased risk of hypersensitivity	OR 5.00 (2.43-10.29), Yes (p = 1.22x10^{-5})	
Asparaginase	Retrospective study	284	Acute Lymphoblastic Leukemia	Hypersensitivity	*HLA-DQA1* *HLA-DRB1* *HLA-DQB1*	*HLA-DRB1*07:01* (rs28724121)	Increased risk of hypersensitivity	OR 2.2 (1.2-4.3), Yes (p = 0.01)	(Gagne, St-Onge et al. 2020)
						*HLA-DRB1*07:01-HLA-DQB1*02:02 haplotype*	Increased risk of hypersensitivity	OR 2.6 (1.3-5.2), Yes (p = 0.006)	
Asparaginase	GWAS Retrospective case control study	5185	Acute Lymphoblastic Leukemia	Pancreatitis	NA	Sixteen *CPA2 SNPs*, highest association with rs199695765	Increased risk of pancreatitis	Yes (p = 9.0x10^{-9})	(Liu, Yang et al.

Table 1: Overview of recent studies (from 2016 onward) analyzing the association between genetic variants and toxicities (and/or other variables)

									2016)
Asparaginase	GWAS Retrospective case control study	1285	Acute Lymphoblastic Leukemia	Pancreatitis	NA	Fourteen SNPs in *ULK2*, highest association with rs281366 C/T allele	Increased risk of pancreatitis	OR 6.71 (3.8-14.2), Yes (p = 5.84x10^{-7})	(Wolthers, Fr et al. 2017)
						RGS6 (rs17179470) G/T allele	Increased risk of pancreatitis	OR 4.39 (2.41-7.96), Yes (p = 1.27x10^{-6})	
						CPA2 (rs2178158)	Increased risk of pancreatitis	OR 1.8 (1.01-3.2), Yes (p = 0,049)	
						CPA2 (rs199695765)	Unknown	No (p > 0.05)	
Asparaginase	GWAS Retrospective case control study	1564	Acute Lymphoblastic Leukemia	Pancreatitis	NA	*PRSS1-2* (rs13228878) G allele	Decreased risk of pancreatitis	OR 0.61 (0.5-0.76), Yes (p = 7.1 x 10^{-6})	(Wolthers, Frandsen et al. 2019)
						PRSS1-2 (rs10273639) T allele	Decreased risk of pancreatitis	OR 0.62 (0.5-0.77), Yes (p = 1.1 x 10^{-5})	
						See study for other strongly significant SNPs (table 1)	NA	NA	
Asparaginase	GWAS	373	Acute Lymphoblastic Leukemia	Hepatotoxicity	NA	Strongest association with *PNPLA3* (rs738409)	Higher alanine aminotransferase (ALT) levels	Yes (p = 2.5 x 10^{-8})	(Liu, Fern et al. 2017)
**METHOTREXATE**									
Methotrexate	Pilot study	109	Acute Lymphoblastic Leukemia	MTX levels, leukopenia and mucositis	*MDR1* *MTHFR*	*MDR1* (rs1045642) CC genotype	Decreased risk of leukopenia grade 1	OR 0.32 (0.14-0.72), Yes (p = 0.007)	(Ramírez-Pacheco, Moreno-Guerrero et al. 2016)
						MDR1 (rs1045642) TT genotype	Increased risk of leukopenia 1	OR 2.36 (1.02-5.48), Yes (p = 0.044)	
						MTHFR (rs1801133) CC genotype	Increased risk of mucositis	OR 3.61 (1.42-9.14), Yes (p = 0.007)	
Methotrexate	Retrospective study	59	Osteosarcoma	Pharmacokinetics and hepato-myelotoxicity	*ABCB1* *ABCC1* *ABCC2* *ABCC3*	*NR1I2* (rs3732361)	Decreased risk of hepatic toxicity	OR 1 (0.01-0.7), Yes (p = 0.014)	(Hegyi, Arany et al. 2017)
						NR1I2 (rs3814058)	Decreased risk of hepatic toxicity	OR 0.3 (0.1-0.7), Yes (p = 0.007)	

Table 1: Overview of recent studies (from 2016 onward) analyzing the association between genetic variants and toxicities (and/or other variables)

					ABCC10 *ABCG2* *GGH* *SLC19A1* *NR1I2*	*NR1I2* (rs6785049)	Decreased risk of hepatic toxicity	OR 0.1 (0.01-0.7), Yes (p = 0.02)	
						ABCC2 (rs2273697)	Increased risk of myelotoxicity	OR 3.3 (1.2-9.4) Yes (p = 0.02)	
						ABCC2 (rs3740066)	Decreased risk of myelotoxicity	OR 0.4 (0.2-0.9), Yes (p = 0.02)	
						NR1I2 (rs3732361)	Decreased risk of myelotoxicity	OR 0.1 (0.01-0.7), Yes (p = 0.013)	
						NR1I2 (rs3814058)	Decreased risk of myelotoxicity	OR 0.3 (0.1-0.7), Yes (p = 0.007)	
						NR1I2 (rs6785049)	Decreased risk of myelotoxicity	OR 0.09 (0.01-0.7), Yes (p = 0.01)	
Methotrexate	Retrospective study	322	Acute Lymphoblastic Leukemia	Pharmacokinetics and mucositis	*SLCO1B1* *SLC19A1* *ABCB1* *ABCG2*	*SLCO1B1* (rs11045879) *SLCO1B1* (rs4149056) *SLCO1B1* (rs2306283) *SLCO1B1* (rs10841753) *SLC19A1* (rs1051266) *SLC19A1* (rs3788200) *SLC19A1* (rs1131596) *SLC19A1* (rs2838958) *ABCB1* (rs1128503) *ABCB1* (rs1045642) *ABCG2* (rs2231137)	Unknown	No (p > 0.05)	(Liu, Gao et al. 2017)
Methotrexate	Retrospective study	141	Acute Lymphoblastic Leukemia	Pharmacokinetics and various toxicities	*SLCO1A2*	miR in *SLCO1A2* (rs4149009) GG genotype	Delayed elimination	Yes (p = < 0.05)	(Wang, Zeng et al. 2018)
						SLCO1A2 (rs4149009) AA, GA, and GG genotypes	Unknown	No (p > 0.05)	

Table 1: Overview of recent studies (from 2016 onward) analyzing the association between genetic variants and toxicities (and/or other variables)

Methotrexate	Retrospective study	37	Osteosarcoma	Pharmacokinetics and various toxicities	*ATIC* *MTHFR* *MTHFR* *SLC19A1*	*ATIC* (rs2372536), *MTHFR* (rs1801131)	Unknown (for MTX concentration)	No ($p > 0.05$)	(Park and Shin 2016)
						SLC19A1 (rs1051266)	Lower plasma MTX levels	Yes ($p = 0.03$)	
						SLC19A1 (rs1051266) GA/AA genotype	Decreased risk of mucositis	OR 0.06 (0.01-0.69), Yes ($p = 0.026$)	
						ATIC (rs2372536) *MTHFR* (rs1801133) *MTHFR* (rs1801131)	Unknown (for toxicities)	No ($p = > 0.05$)	
Methotrexate	Retrospective study	88	Acute lymphoblastic leukemia and non Hodgkin malignant lymphoma	Leukopenia, thrombocytopenia mucositis, neurotoxicity	*SLC19A1*	*SLC19A1* (rs2838958) TC/CC genotype	Decreased risk of mucositis	OR 0.226 (0.071-0.725), Yes ($p = 0.009$)	(Kotnik, Jazbec et al. 2017)
Methotrexate	Retrospective study	196	Osteosarcoma	Hepatotoxicity	Germline polymorphisms of 31 genes relevant for transport or metabolism	*ABCB1* (rs1128503) C allele	Increased risk of hepatotoxicity	OR 2.21 (1.22-4.00), Yes ($p = 0.009$)	(Hattinger, Biason et al. 2016)
						ABCC2 (rs2273697) G allele	Increased risk of hepatotoxicity	OR 2.48 (1.47-4.18), Yes ($p = 0.001$)	
						GGH (rs1800909) C allele	Increased risk of hepatotoxicity	OR 2.93 (1.53-5.62), Yes ($p = 0.001$)	
						ABCC2 (rs3740066) G allele	Increased risk of nausea/vomiting	OR 3.15 (1.06-9.37), Yes ($p = 0.039$)	
Methotrexate	Retrospective and prospective study	64	Acute lymphoblastic leukemia	Hematotoxicity	*DHFR* *MTHFR* *DPYD* *TYMS*	*DHFR* (rs70991108) insertion/insertion genotype	Increased risk of leucopenia	OR 5.4 (1.6-17.8), Yes ($p = 0.006$)	(Yousef, Farhad et al. 2019)
						MTHFR (rs1801133) CC/CT genotype	Increased risk of leucopenia	OR 4.5 (1.2-17.0), Yes ($p = 0.03$)	
						MTHFR (rs1801131) CC/AC genotype	Increased risk of neutropenia	OR 6.1 (1.3-29.5), Yes ($p = 0.04$)	

Table 1: Overview of recent studies (from 2016 onward) analyzing the association between genetic variants and toxicities (and/or other variables)

						TYMS (rs151264360) deletion/deletion and deletion/insertion genotype	Increased risk of neutropenia	OR 6.0 (1.2-31.1), Yes (p = 0.04)	
Methotrexate	Prospective study	35	Acute lymphoblastic leukemia	Hepatotoxicity, gastrointestinal toxicity, mucositis, neurotoxicity, skin toxicity, hematotoxicity, renal toxicities, phlebitis	*MTHFR*	*MTHFR* (rs1801133) CT genotype	Increased mean global toxicity score	Yes (p = 0.002)	(Mahmoud, Mdhaffar et al. 2018)
Methotrexate	Retrospective study	56	Acute lymphoblastic leukemia and lymphoma	Leukoencephalopathy, neurotoxicity	*ATIC* *ADORA2A* *MTHFR* *ABCB1*	*ADORA2A* (rs2298383) CC genotype	Increased risk of leukoencephalopathy	OR 15.30 (2.43-96.6), Yes (p = 0.004)	(Tsujimoto, Yanagimachi et al. 2016)
Methotrexate	Prospective study	117	Acute lymphoblastic leukemia	Mucositis	*CNOT4* *miR-1206* *miR-2053*	*miR-1206* (rs2114358) AA/AG genotype	Increased risk of mucositis	OR 3.58 (1.12-11.46), Yes (p = 0.024)	(Guttierez-Camino, Oosterom et al. 2016)
						CNOT4 (rs3812265) *miR-2053* (rs10505168)	Unknown	No (p = > 0.05)	
Methotrexate	Prospective study	38	Acute lymphoblastic leukemia	Various toxicities	*ARID5B* *ABCC2* *SLCO1B1* *MTHFR*	*ABCC2* (rs717620) CT/TT genoype	Possible increased risk of leukopenia grade 1-4	NA, Yes (p =0.03)	(Razali, Noorizhab et al. 2020)
						ARID5B (rs4948496) TC/CC genotype	Possible increased risk of leukopenia grade 1-4	NA, Yes (p =0.02)	
						SLCO1B1 (rs4149056) *MTHFR* (rs1801133)	Unknown	No (p > 0.05)	
**PLATINUM COMPOUNDS**									

Table 1: Overview of recent studies (from 2016 onward) analyzing the association between genetic variants and toxicities (and/or other variables)

Cisplatin	Retrospective study	149	Mixed	Ototoxicity	*ACYP2*<br>*TPMT*<br>*COMT*	*ACYP2* (rs1872328) GG genotype	Higher frequency of ototoxicity	Yes (p = 0.027)	(Thiesen, Yin et al. 2017)
						TPMT (rs12201199)<br>*TPMT* (rs1142345)<br>*TPMT* (rs1800460)<br>*COMT* (rs4646316)<br>*COMT* (rs9332377)	Unknown	No (p > 0.05)	
Cisplatin	Retrospective study	156	Osteosarcoma	Ototoxicity	*ACYP2*	*ACYP2* (rs1872328) GG/AG genotype	Higher frequency of ototoxicity	Yes (p = 0.027)	(Vos, Guchelaar et al. 2016)
Cisplatin and carboplatin	Prospective study	61	Mixed	Ototoxicity	*MT-RNR1*<br>*GJB2*<br>*GSTP1*	*GSTP1* (rs1695)	Increased risk of cisplatin-induced ototoxicity	OR 10.667(2.657-42.826), Yes (p = 0.001)	(Liberman, Goffi-Gomez et al. 2018)
						GJB2 (rs80338939)<br>*MT-RNR1* (rs267606617)	Unknown	No (NA)	
Cisplatin	Epigenome-wide association study	62	Medulloblastoma and primitive neuroectodermal tumor	Ototoxicity	NA	Differential DNA methylation in *PAK4* (cg14010619)	Increased risk of ototoxicity	Yes (p = 0.001)	(Brown, Foster et al. 2017)
Cisplatin	Prospective study	60	Mixed	Ototoxicity	*ERCC1*<br>*ERCC2*<br>*XRCC1*	*ERCC1* (rs25487)<br>*ERCC2* (rs13181)<br>*XRCC1* (rs11615)	Unknown	No (p > 0.05)	(Turan, Kantar et al. 2019)
**GLUCOCORTICOSTEROIDS**									
Prednisone and/or dexamethasone	Retrospective study	346	Acute lymphoblastic leukemia	Hepatotoxicity, glucose metabolism abnormalities, central nervous system and/or behavior abnormalities,	*N363S*<br>*ER22/23EK*<br>*Bcl-1 GCR*	*N363S* carriers	Increased risk of hepatotoxicity	OR 3.6 (1.59-8.28), Yes (p = 0.004)	(Eipel, Hegyi et al. 2016)
						N363S carriers	Increased risk of glucose metabolism abnormalities	OR 8.8 (2.85-27.37), Yes (p = 0.001)	
						ER22/23EK<br>*Bcl-1 GCR*	Unknown	No (p > 0.05)	

Table 1: Overview of recent studies (from 2016 onward) analyzing the association between genetic variants and toxicities (and/or other variables)

				hypertension					
Corticosteroids	Retrospective study	615	Acute lymphoblastic leukemia	Bone toxicity	*ABCB1*<br>*BDNF*<br>*COMT*<br>*DHFR*<br>*HFE*<br>*IL1B*<br>*MTHFR*<br>*MTR*<br>*NOS3*<br>*NR3C1*<br>*PAI-1*<br>*RFC1*<br>*SLC6A4*<br>*SLCO2A1*<br>*TCN2*<br>*TS*<br>*VitDR*	*2R/2R TS* genotype	Increased risk of osteonecrosis	HR 2.71 (1.23-5.96), Yes (p = 0.13)	(Finkelstein, Blonquist et al. 2017)
						2R/2R TS genotype	Increased risk of bone fracture	HR 2.10 (1.11-3.96), Yes (p = 0.022)	
Prednisone and/or dexamethasone	Retrospective study	304	Acute lymphoblastic leukemia	Osteonecrosis	*BCL2L11*	*BCL2L11* (rs2241843) TT genotype	Higher cumulative incidence	HR 2.4 (1.2-4.8), Yes (p = 0.01)	(Plesa, Gagné et al. 2019)
						BCL2L11 (rs724710) CC genotype	Higher cumulative incidence in high-risk group	HR 5.7 (1.6-21.9), Yes (p = 0.008)	
						BCL2L11 (rs2241842, rs73954926, rs72836346, rs7582030, rs72836345, rs6750142)	Unknown	No (p > 0.05)	
**THIOPURINES**									
6-Mercaptopurine	Retrospective study	404	Acute lymphoblastic leukemia	Maximum tolerable dose and treatment	*TPMT*<br>*NUDT15*	*NUDT15* (rs116855232) TT, TC and CC genotype	Lowest tolerance (TT genotype) to highest tolerance (CC	Yes (p = 0.0001)	(Liang, Yang et al.

Table 1: Overview of recent studies (from 2016 onward) analyzing the association between genetic variants and toxicities (and/or other variables)

				outcome			genotype)		2016)
						TPMT (rs1142345) AG and AA genotype	Lowest tolerance (AG genotype) to highest tolerance (AA genotype)	Yes (p = 0.034)	
						TPMT (rs1142345) *NUDT15* (rs116855232) homozygote and heterozygote genotypes	No differences in treatment outcome	No (p > 0.05)	
6-Mercaptopurine	Retrospective study	68	Acute lymphoblastic leukemia	Leukopenia and hepatotoxicity	*TPMT* *ITPA* *ABCC4* *ABCB1*	*ABCB1* GT and TT genotype	Higher frequency of hepatotoxicity	Yes (p = 0.030)	(Milosevic, Kotur et al. 2018)
						ITPA *ABCC4*	Unknown	No (p > 0.05)	
6-Mercaptopurine	Retrospective study	105	Acute lymphoblastic leukemia	Leukopenia and hepatotoxicity	*NUDT15*	*NUDT15* (rs116855232) CT and TT genotype	Increased risk of leukopenia and early-onset leukopenia	OR 3.617 (1.377-9.051), Yes (p = 0.009)	(Zhou, Li et al. 2018)
						NUDT15 (rs116855232) CT and TT genotype	Increased risk of early-onset leukopenia	OR 9.63 (2.764-33.514), Yes (p = $3.75x10^{-4}$)	
6-Mercaptopurine	Follow-up study	124	Acute lymphoblastic leukemia	Leukopenia	*TPMT* *ITPA* *NUDT15*	One or more risk alleles: *TPMT *2, *3A, *3B and *3C* (resp. rs1800462, rs1800460 and rs1142345) and/or *NUDT15 *2, *3, *4 and *6* (rs116855232, rs55440599 and rs147390019)	Increased risk of early-onset leukopenia grade 3-4	OR 3.194 (1.075-9.483), Yes (p = 0.037)	(Soler, Olano et al. 2018)

Table 1: Overview of recent studies (from 2016 onward) analyzing the association between genetic variants and toxicities (and/or other variables)

						One or more risk alleles: *TPMT *2, *3A, *3B and *3C* (resp. rs1800462, rs1800460 and rs1142345) and/or *NUDT15 *2, *3, *4 and *6* (rs116855232, rs55440599 and rs147390019)	Increased risk of late-onset leukopenia grade 3-4	OR 3.625 (1.163-11.304), Yes (p = 0.026)	
6-Mercaptopurine	Prospective study	63	Acute lymphoblastic leukemia	Myelotoxicity	*TPMT* *ITPA* *NUDT15*	One risk allele: *TPMT *2, *3A, *3B and *3C* (resp. rs1800462, rs1800460 and rs1142345) and/or *NUDT(415CT)* and/or *ITPA(198CA)*	Increased risk of late-onset neutropenia	OR 6.2 (1.7-21.8), Yes (p = 0.003)	(Khera, Trehan et al. 2019)
6-Mercaptopurine	Retrospective study	82	Acute lymphoblastic leukemia	Myelotoxicity	*ITPA* *NUDT15*	*NUDT15* (rs116855232) CT/ TT genotype	Increased risk of myelotoxicity	OR 7.44 (1.3-42.63), Yes (p = 0.01)	(Chiengthong, Ittiwut et al. 2016)
						ITPA (rs1127354)	Unknown	No (p > 0.05)	
6-Mercaptopurine	Retrospective study	Mixed: 180<br>ALL: 689	Mixed cohort and acute lymphoblastic leukemia	Hematotoxicity	*TPMT* *NUDT15*	*NUDT15* (rs746071566)	Increased frequency of hematotoxicity	Yes (p = $4x10^{-16}$)	(Schaeffeler, Jaeger et al. 2019)
						NUDT15 (rs116855232)	Increased frequency of hematotoxicity	Yes (p = $1.5x10^{-12}$)	
						NUDT15 (rs869320766)	Increased frequency of hematotoxicity	Yes (p = 0.05)	
6-Mercaptopurine	Retrospective study	305	Acute lymphoblastic leukemia	Hematotoxicity	*TPMT* *PACSIN2* *ITPA* *MTHFR* *MTRR* *MTHFD1*	*PACSIN2* (rs2413739) TT genotype	Increased risk of hematotoxicity	OR 2.48 (1.02-5.91), Yes (p = 0.041)	(Smid, Karas-Kuzelicki et al. 2016)
						*TPMT*1*3*	Increased risk of hematotoxicity	OR 51.69 (15.24-219.76), Yes (p = $3.89x10^{-9}$)	

Table 1: Overview of recent studies (from 2016 onward) analyzing the association between genetic variants and toxicities (and/or other variables)

					BHMT<br>*GNMT*	*GNMT* (rs10948059) CT/TT genotype	Decreased risk of hematotixicity	OR 0.43 (0.19-0.99), Yes (*p* = 0.045)	
						MTHFR (rs1801133) CT/TT genotype and/or *MTHFR* (rs1801131) AA/AC genotype	Increased risk of hematotoxicity	OR 10.41 (1.72-109.84), Yes (*p* = 0.025)	
						ITPA (rs1127354)<br>*ITPA* (rs7270101)<br>*MTRR* (rs1801394)<br>*MTHFD1* (rs2236225)<br>*BHMT* (rs3733890)	Unknown	No (*p* > 0.05)	
						*TPMT (*rs1800460) and *TPMT* (rs1142345) in combination with *PACSIN2* (rs2413739)	Increaser risk of hematotoxicity when carrying *TPMT* wild or variant type and *PACSIN2* (rs2413739) TT genotype	NA, Yes (*p* = 0.006)	
6-Mercaptopurine	Retrospective study	185	Acute lymphoblastic leukemia	Neutropenia	211 pharmacogenetic-related genes	*ABCC4* (rs3765534) CT/TT genotype	Increased risk of neutropenia	OR 6.36 (1.43-58.73), Yes (*p* = 0.006)	(Kim, Seo et al. 2018)
						APEX1 (rs2307486) G variant allele	Increased risk of early onset neutropenia	OR 3.44 (1.04-9.89), Yes (*p* = 0.02)	
						ABCC4 (rs3765534), *APEX1* (rs2307486) and *NUDT15* (rs116855232)	Higher cumulative incidence of neutropenia	Yes (resp. *p* = 0.01, <0.01, <0.01)	
6-Mercaptopurine	Retrospective study	41	Acute lymphoblastic leukemia	Hematotoxicity	*SLC19A1*<br>*ABCB1*<br>*ABCC2*<br>*ABCC4*<br>*ABCG2*	*ABCB1* CC genotype	Higher incidence of neutropenia grade 1-2	Yes (*p* = 0.023)	(Gervasini, de Murillo et al. 2017)
						ABCB1 TT genotype	Higher incidence of thrombopenia grade 1-2	Yes (*p* = 0.004)	
						ABCC4 CA genotype	Higher incidence of anemia grade 1-2	Yes (*p* = 0.018)	

Table 1: Overview of recent studies (from 2016 onward) analyzing the association between genetic variants and toxicities (and/or other variables)

6-Mercaptopurine	Prospective follow-up study	95	Acute lymphoblastic leukemia	Leukopenia	*NUDT15*<br>*ABCC4*<br>*TPMT*<br>*ITPA*<br>*SLCO1B1*<br>*MTHFR*	*ABCC4* (rs3765534) GG/GA genotype	Increased risk of leukopenia	HR 1.947 (1.19-3.19), Yes (p = 0.008)	(Tanaka, Nakadate et al. 2018)
						NUDT15 (rs116855232) CC/CT genotype	Increased risk of leukopenia	HR 2.79 (1.80-4.31), Yes (p = $4.41x10^{-6}$)	
						NUDT15 (rs186364861) GG/GA genotype	Increased risk of leukopenia	HR 2.29 (1.01-5.12), Yes (p = 0.045)	
6-Mercaptopurine	Retrospective study	151	Acute lymphoblastic leukemia	Neutropenia, febrile neutropenia	*TPMT*<br>*ITPA*<br>*NUDT15*	*ITPA* (rs7270101)<br>*ITPA* (rs1127354)<br>*NUDT15* (rs116855232)	Unknown	No (p > 0.05)	(Wahlund, Nilsson et al. 2020)
						Any TPMT variant:<br>*TPMT* (rs1800462) GC genotype<br>*TPMT* (rs1800460) AG genotype<br>*TPMT* (rs1142345) GA genotype	Decreased risk of neutropenia in maintenace phase	HR 0.49 (0.27-0.89), Yes (p = 0.019)	
**TOPOISOMERASE I AND II INHIBITORS**									
Irinotecan, etoposide	Retrospective case control study	102	Medulloblastoma	Various toxicities	*MSH2*<br>*RAD50*<br>*NBN*<br>*FANCM*<br>*ERCC2*<br>*EXO1*	*NBN* variants: p.Lys219fs*19/*NBN* and p.I171V/*NBN*	Experienced a rare grade 4 adverse events±	Yes (p = 0.001)	(Trubicka, Zemojtel et al. 2017)
						MSH2, RAD50 and NBN variants:<br>p.V606I/*MSH2*<br>p.A733T/*MSH2*<br>p.R1093* /*RAD50*<br>p.Lys219fs*19/*NBN*<br>p.I171V/*NBN*	Experienced a rare grade 4 adverse events±	Yes (p = 0.0005)	

Table 1: Overview of recent studies (from 2016 onward) analyzing the association between genetic variants and toxicities (and/or other variables)

						Variants in *FANCM, ERCC2 and EXO1:* p.L694*/*FANCM* p.R695C/*ERCC2* p.V738L/*EXO1*	Three patients experienced rare grade 4 adverse events± and carried these variants	NA	
**VINCA ALKALOIDS**									
Vincristine	Retrospective study	508	Acute lymphoblastic leukemia	Gastrointestinal, hepatic and neurological toxicities	28 single-nucleotide polymorphisms (SNPs) and 2 gene deletions involved in efficacy and adverse effects	*ITPA* (rs1127354)	Increased risk of neurotixicity	OR 13.23 (1.74-100.65), Yes (before correction *p* = 0.013), No after correction (*p* > 0.05)	(Franca, Rebora et al. 2017)
						ITPA (rs1127354)	Increased risk of gastrointestinal toxicity	OR 7.73 (1.04-57.34), Yes (before correction *p* = 0.046), No after correction (*p* > 0.05)	
						ADORA2A (rs2236624)	Increased risk of hepatic toxicity	OR 2.25 (1.03-4.88), Yes (before correction *p* = 0.041), No after correction (*p* > 0.05)	
						ABCC1 (rs246240)	Increased risk of neurotoxicity	OR 4.61 (1.12-19.02), Yes (before correction *p* = 0.035), No after correction (*p* > 0.05)	

Table 1: Overview of recent studies (from 2016 onward) analyzing the association between genetic variants and toxicities (and/or other variables)

Vincristine	Retrospective study	239	Acute lymphoblastic leukemia	Neurotoxicity	*CYP3A5* *CEP72*	*CYP3A5*1/*3/*6/*7, CEP72* CC, CT, TT genotype	Unknown	No ($p > 0.05$)	(McClain, Bernhardt et al. 2016)
Vincristine	Prospective cohort study	78	Mixed	Peripheral neuropathy	*CYP3A5* *MDR1* *MAPT*	*CYP3A5*1/*3/*6/*7*	Unknown	No ($p > 0.05$)	(Skiles, Chiang et al. 2018)
						*MDR1*and *MAPT* polymorphisms (not specified)	Unknown	No ($p > 0.05$)	
Vincristine	Retrospective study	167	Acute lymphoblastic leukemia	Peripheral neuropathy	*CEP72*	*CEP72* (rs924607) TT genotype	Increased risk of peripheral neuropathy	OR 3.43 (1.15-10.3), Yes ($p = 0.02$)	(Wright, Amstutz et al. 2019)
Vincristine	Retrospective study	152	Acute lymphoblastic leukemia	Neurotoxicity	150 SNPs involved in vincristine pharmacokinetics and 13 microRNAs	*ABCC2* (rs3740066) AG genotype	Decreased risk of neurotoxicity grade 1-4	OR 0.23 (0.10-0.53), Yes (after FDR correction $p = 0.0036$)	(Lopez-Lopez, Gutierrez-Camino et al. 2016)
						ABCC2 (rs12826) AG genotype	Decreased risk of neurotoxicity grade 1-4	OR 0.24 (0.10-0.54), Yes (after FDR correction $p = 0.0036$)	
						ABCC2 (rs3740066) AG genotype	Decreased risk of neurotoxicity grade 1-2	OR 0.15 (0.06-0.43), Yes (after FDR correction $p = 0.016$)	
						ABCC2 (rs12826) AG genotype	Decreased risk of neurotoxicity grade 1-2	OR 0.15 (0.05-0.41), Yes (after FDR correction $p = 0.016$)	
						ABCC2 (rs3740066) GG genotype and *ABCC2* (rs12826) GG genotype	No increased risk of neurotoxicity grade 3-4	No (after FDR correction $p > 0.05$)	
						*ABCC2 (*rs2756109-rs2273697-rs2073337-	Higher frequency of neurotoxicity grade 1-4 and grade 1-2	NA, Yes (before FDR correction $p = > 0.05$), No after	

Table 1: Overview of recent studies (from 2016 onward) analyzing the association between genetic variants and toxicities (and/or other variables)

						rs4148394-rs4148396) GGAAC haplotype		FDR correction *(p* > 0.05)	
						ABCC2 (rs3740066-rs3740065-rs12826-rs12762549-rs11190298) ATAGG haplotype	Lower frequency of neurotoxicity grade 1-4 and grade 1-2	NA, Yes (before FDR correction *p* = > 0.05), No after FDR correction *(p* > 0.05)	
						ABCC1 (rs3784862-rs246240-rs875740-rs11642957-rs3784864-rs11075293) AATCGG haplotype	Lower frequency of neurotoxicity grade 1-4	NA, Yes (before FDR correction *p* = > 0.046), No after FDR correction *(p* > 0.05)	
Vincristine	Retrospective study	133	Acute lymphoblastic leukemia	Peripheral neuropathy	*ABCB1* *ABCC2* *CEP72* *ETAA1* *MTNR1B*	*ABCB1* (rs1045642) *ABCB1* (rs1128503) *ABCC2* (rs717620) *CEP72* (rs924607) *MTNR1B* (rs12786200)	Unknown	No (*p* > 0.05)	(Zgheib, Ghanem et al. 2018)
Vincristine	Retrospective study	179	Acute lymphoblastic leukemia	Peripheral neurotoxicity	SNPs in microRNAs	hsa-mir-3117 (rs12402181) AG genotype	Decreased risk of neurotoxicity grade 1-4	OR 0.16 (0.05-0.55), Yes (*p* = 0.00042), No after FDR correction *(p* > 0.05)	(Gutierrez-Camino, Umerez et al. 2017)
						hsa-mir-6076 (rs35650931) CC genotype	Decreased risk of neurotoxicity grade 1-4	OR 0.22 (0.05-0.97), Yes (*p* = 0.017), No after FDR correction *(p* > 0.05)	
						hsa-mir-4481 (rs7896283) CC genotype	Increased risk of neurotoxicity grade 1-4	OR 2.62 (1.15-5.95), Yes (*p* = 0.017), No after FDR correction *(p* > 0.05)	

Table 1: Overview of recent studies (from 2016 onward) analyzing the association between genetic variants and toxicities (and/or other variables)

						See study for 9 other SNPs with significant results	NA	NA	
Vincristine	Retrospective study	142	Acute lymphoblastic leukemia	Peripheral neuropathy	*CEP72* (rs924607) TT genotype	*CEP72* (rs924607) TT genotype	Unknown	No (*p* > 0.05)	(Gutierrez-Camino, Martin-Guerrero et al. 2016)
Vincristine	GWAS	1795 (two cohorts)	Acute lymphoblastic leukemia	Peripheral neuropathy	NA	SNP rs1045644	Decreased risk of neuropathy grade 3-4	HR 0.27 (0.16-0.50), Yes (p = 8.66x10^{-6} (ADVANCE trail), 8.65x10^{-7} (POG cohort)	(Li, Sajdyk et al. 2019)
						SNP rs7963521	Increased risk of neuropathy grade 3-4	HR 2.23 (NA), Yes (p = 1.05x10^{-5})	
Vincristine	Retrospective study	152	Acute lymphoblastic leukemia	Neurotoxicity	21 SNPs in *TUBB1, TUBB2A, TUBB2B, TUBB3, TUBB4* *MAPT* miRNAs	*MAPT* (rs11867549) AA genotype	Increased risk of neurotoxicity grade 1-2	OR 4.40 (0.83-23.43), Yes (before FDR *p* = 0.09), No (after FDR correction *(p* > 0.05))	(Martin-Guerrero, Gutierrez-Camino et al. 2019)
						MAPT (rs11867549) AG/GG genotype	Decreased risk of neurotoxicity grade 3-4	OR 0.21 0.04-0.96), Yes (before FDR *p* = 0.020), No (after FDR correction *(p* > 0.05))	
						Mir-202 (rs12355840) CT/CC genotype	Increased risk of neurotoxicity grade 1-2	OR 2.88 (1.07-7.72), Yes (before FDR *p* = 0.032), No (after FDR correction *(p* > 0.05))	
						TUBB3 (rs4395073-rs4558416) CA	Higher frequency neurotoxicity grade	NA, Yes (before FDR *p* = 0.032),	

Table 1: Overview of recent studies (from 2016 onward) analyzing the association between genetic variants and toxicities (and/or other variables)

						haplotype	1-2	No (after FDR correction (p > 0.05))	
						TUBB3 (rs4395073-rs4558416) TH haplotype	Lower frequency of neurotoxicity grade 1-2	NA, Yes (before FDR p = 0.041), No (after FDR correction (p > 0.05))	
						MAPT (rs1001945-rs11867549) CG haplotype	Lower frequency of neurotoxicity grade 3-4	NA, Yes (before FDR p = 0.038), No (after FDR correction (p > 0.05))	
Vincristine	WES screening Retrospective study	237	Acute lymphoblastic leukemia	Peripheral neuropathy	NA	*SYNE2* (rs2781377) GA/AA genotype	Increased risk of neuropathy	OR 2.5 (1.2-5.2), Yes (p = 0.01)	(Abaji, Ceppi et al. 2018)
						MRPL47 (rs10513762) CT/TT genotype	Increased risk of neuropathy	OR 3.3 (1.4-7.7), Yes (p = 0.01)	
						BAHD1 (rs3803357) CA/AA genotype	Decreased risk of neuropathy	OR 0.35 (0.2-0.7), Yes (p = 0.007)	

Note: SNPs: single nucleotide polymorphism; FDR: false discovery rate; OR: Odds ratio; HR: Hazard ratio; NA: Not applicable; MTX: methotrexate;

Unknown effect: some genetic variant did not reach significance (p > 0.05). In most studies there were not enough patients carrying this variant (leading to low or no power) and it was not possible to analyze the association of this variant with toxicities. Therefore, we used the term "unknown effect" when no significance was reached.

#: see supplemental material of Nava et al. (19) for gene expression (poor, rapid or normal metabolizer)

±: include pneumonia, central nervous system toxicity, colitis with gastrointenstinal bleeding, enterocolitis
